# Dynamic transition of Tregs to cytotoxic phenotype amid systemic inflammation in Graves’ ophthalmopathy

**DOI:** 10.1172/jci.insight.181488

**Published:** 2024-11-22

**Authors:** Zhong Liu, Shu-Rui Ke, Zhuo-Xing Shi, Ming Zhou, Li Sun, Qi-Hang Sun, Bing Xiao, Dong-Liang Wang, Yan-Jin Huang, Jin-Shan Lin, Hui-Shi Wang, Qi-Kai Zhang, Cai-Neng Pan, Xuan-Wei Liang, Rong-Xin Chen, Zhen Mao, Xian-Chai Lin

**Affiliations:** State Key Laboratory of Ophthalmology, Zhongshan Ophthalmic Center, Guangdong Provincial Key Laboratory of Ophthalmology and Visual Science, Sun Yat-sen University, Guangzhou, China.

**Keywords:** Autoimmunity, Genetics, Autoimmune diseases, Bioinformatics

## Abstract

Graves’ disease (GD) is an autoimmune condition that can progress to Graves’ ophthalmopathy (GO), leading to irreversible damage to orbital tissues and potential blindness. The pathogenic mechanism is not fully understood. In this study, we conducted single-cell multi-omics analyses on healthy individuals, patients with GD without GO, newly diagnosed patients with GO, and treated patients with GO. Our findings revealed gradual systemic inflammation during GO progression, marked by overactivation of cytotoxic effector T cell subsets, and expansion of specific T cell receptor clones. Importantly, we observed a decline in the immunosuppressive function of activated Treg (aTreg) accompanied by a cytotoxic phenotypic transition. In vitro experiments revealed that dysfunction and transition of GO-autoreactive Treg were regulated by the yin yang 1 (*YY1*) upon secondary stimulation of thyroid stimulating hormone receptor (TSHR) under inflammatory conditions. Furthermore, adoptive transfer experiments of the GO mouse model confirmed infiltration of these cytotoxic Treg into the orbital lesion tissues. Notably, these cells were found to upregulate inflammation and promote pathogenic fibrosis of orbital fibroblasts (OFs). Our results reveal the dynamic changes in immune landscape during GO progression and provide direct insights into the instability and phenotypic transition of Treg, offering potential targets for therapeutic intervention and prevention of autoimmune diseases.

## Abstract 2

Autoimmune Treg transition into a cytotoxic phenotype under high-inflammatory conditions through YY1 regulation, leading to the development of GO.

## Graphical abstract

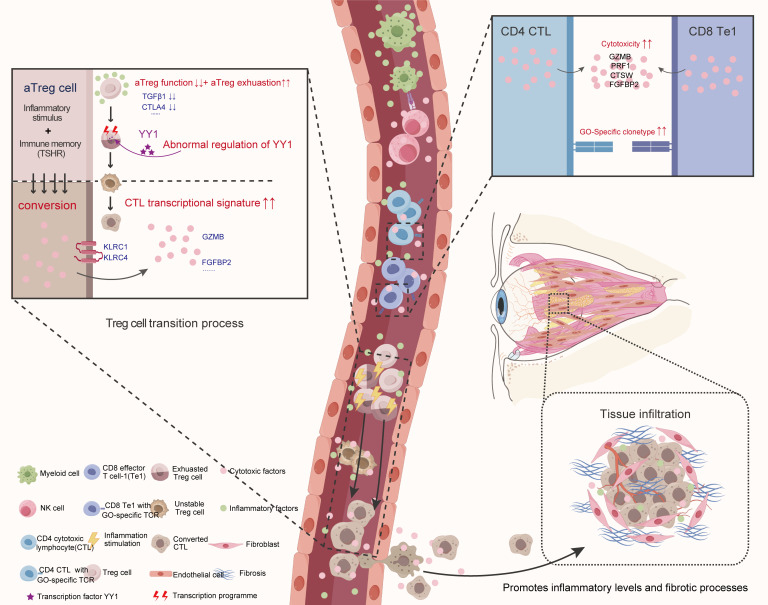

## Introduction

Graves’ disease (GD) is an autoimmune disorder primarily characterized by an enlarged and overactive thyroid gland (Graves’ hyperthyroidism [GH]) (1). As GD progresses, approximately 20%–50% of patients develop Graves’ ophthalmopathy (GO), marked by disfiguring and debilitating symptoms such as eyelid retraction, proptosis, or optic neuropathy (2). Factors such as age, sex, smoking habits, high titers of thyroid stimulating hormone receptor (TSHR) autoantibodies, and the degree of initial inflammation are recognized as risk factors for the development of GO in individuals with GD (2). The first-line treatment for GO, intravenous glucocorticoids, has limited efficacy, carries the risk of multiple side effects, and may lead to relapse upon cessation, which necessitates surgical intervention to alleviate orbital congestion and prevent vision deterioration (1, 3). Despite ongoing clinical trials exploring various treatments, such as lymphocyte proliferation inhibitors or IGF-IR antagonists, many patients do not achieve complete symptom remission and continue to suffer from a reduced quality of life (1, 4, 5). Thus, a deeper understanding of GO’s pathogenesis is crucial for enhancing current treatments and discovering therapeutic targets.

CD4 T cells are central to the inflammatory progression and tissue infiltration in GO (6). The interplay between Th1 and Th2 cells (7, 8), as well as the pathogenic role of Th17 cells (9, 10), is well established in GO’s advancement. CD4 cytotoxic T lymphocytes (CTLs), known for their potent response to autoantigens, are believed to contribute to the initiation and progression of GO (4). CD4 T cells stimulate orbital fibroblasts (OFs), the primary target cells in GO, to produce hyaluronic acid and differentiate into adipocytes and myofibroblasts (MYFs), and this leads to the hallmark pathological changes of GO, such as the expansion of orbital soft tissue and fibrosis (1, 11).

Treg, a distinct subset of CD4 T cells, are essential for maintaining immune equilibrium (12). The X-chromosome-encoded forkhead domain protein 3 (*FOXP3*) transcription factor is crucial for the differentiation, stability, and function of Treg (13). Dysfunction of *FOXP3*^+^ Treg is associated with autoimmune pathologies (12). Besides, research has shown that Treg lose their stability and suppressive capacity, shifting toward an effector T cell phenotype within the inflammatory conditions of autoimmune diseases (12, 14). However, the influence of Treg on GO pathogenesis and the associated transcriptional dynamics has yet to be fully elucidated.

In this study, we used single-cell multi-omics sequencing techniques to explore the role of immune cells in the development of GO. Our toolkit included single-cell RNA-Seq (scRNA-Seq), single-cell assay for transposase-accessible chromatin sequencing (scATAC-Seq), and single-cell T cell receptor sequencing (scTCR-Seq). The goal was to dissect the complex immune cell interactions and transformations during GO progression. Our data reveal an increase in inflammatory activity among individuals in newly diagnosed patients with GO, marked by heightened cytotoxic activity in CD4 CTLs and CD8 T effector cells, along with the appearance of antigen clones specific to GO. Furthermore, as GO progresses, activated Treg (aTreg) exhibited a distinct transcriptional signature characteristic of *FGFBP2*^+^*KLRC1*^+^ CTL, accompanied by a decrease in immunosuppressive function, potentially due to the abnormal regulation of the transcription factor *YY1* within the disease-specific inflammatory environments. Adoptive transfer experiments demonstrated that these cells migrated to and infiltrated orbital tissues, potentially contributing to fibrosis and alterations in the extracellular matrix (ECM). Our study highlights a pronounced inflammatory state in patients with GH who develop GO compared with those who do not, which suggests that the escalating inflammation may lead to a detrimental shift in Treg behavior, offering a promising target for therapeutic intervention or even prophylaxis.

## Results

### Construction of single-cell atlas and cell type identification in PBMC.

**Figure 1:**
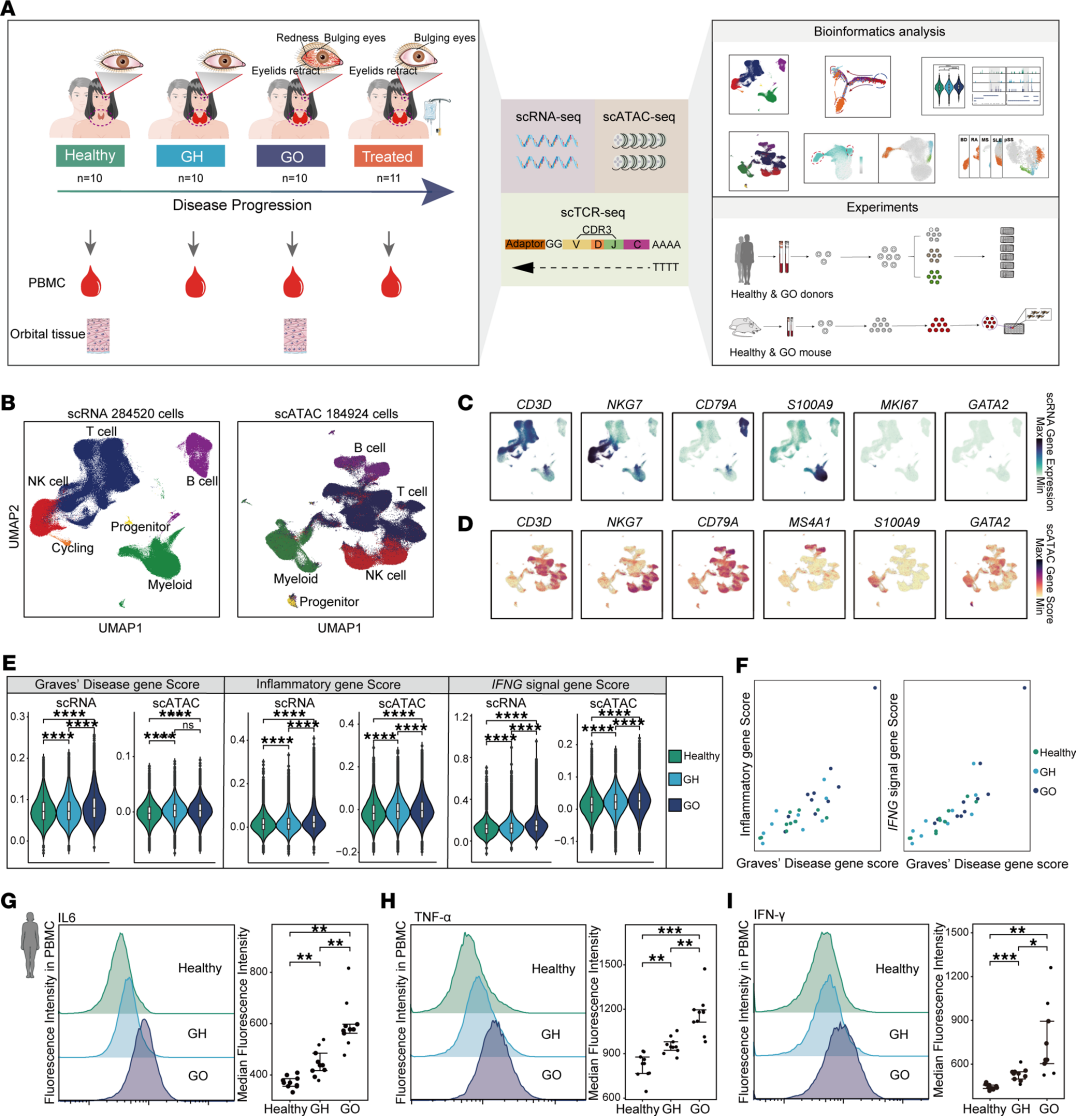
Single-cell atlas of expression and chromatin accessibility revealed enhanced inflammation in the systemic immune environment during GO progression. (**A**) Schematic representation of single-cell multi-omics sequencing (scRNA-Seq, scATAC-Seq, and scTCR-Seq) experimental design. PBMCs were isolated from healthy controls (Healthy, *n* = 10), patients with Graves’ disease without ophthalmopathy (GH, *n* = 10), patients with Graves’ ophthalmopathy (GO, *n* = 10), and corticosteroid-treated patients (Treated, *n* = 11), followed by processing using the 10X Genomics platform. (**B**) UMAP plots depicted major immune cell types in peripheral blood based on scRNA-Seq and scATAC-Seq datasets. (**C** and **D**) UMAP plots illustrated the expression levels (**C**) and gene activity scores (**D**) of typical marker genes for major immune cell types. (**E**) Violin plots showed the expression and activity scores of Graves’ disease, inflammatory, and *IFNG* signal gene sets among Healthy, GH, and GO groups. Data are represented as the median IQR. ****P_adj_ < 0.00001 by Mann-Whitney *U* test. (**F**) Scatter plots depicted the correlation of Graves’ disease gene and inflammatory gene scores across all samples in Healthy, GH, and GO groups. (**G**–**I**) Flow density plots and dot plots displayed the expression levels of IL-6 (**G**), TNF-α (**H**), and IFN-γ (**I**) in peripheral blood from Healthy, GH, and GO donors (Healthy, *n* = 9; GH, *n* = 9; GO, *n* = 9). Data are represented as the median IQR. *P_adj_ < 0.05, **P_adj_ < 0.001, ***P_adj_ < 0.0001 by Mann-Whitney *U* test.

In this study, we analyzed single-cell data from 41 peripheral blood mononuclear cell (PBMC) samples, which included samples from each of the following 4 cohorts: healthy controls, patients with GD without ophthalmopathy (GH), newly diagnosed patients with GO with a clinical activity score (15) (CAS) of 3 or higher, and patients with GO treated with methylprednisolone exhibiting a CAS of less than 3 (Figure 1A). Each sample underwent parallel multi-omics profiling using the 10X Genomics platform, encompassing scRNA-Seq, scATAC-Seq, and scTCR-Seq (Figure 1A). After detailed bioinformatics analysis, we combined findings from human and mouse cell experiments to validate the key molecular characteristics of peripheral blood immune cells during GO progression (Figure 1A). After implementing rigorous quality control measures, we compiled a dataset of 284,520 single-cell transcriptomes (Figure 1B and Supplemental Figure 1A; supplemental material available online with this article; https://doi.org/10.1172/jci.insight.181488DS1) and 184,924 single-cell chromatin accessibility profiles from the 3 nontreated groups (Figure 1B and Supplemental Figure 1B). We utilized Seurat and Signac for clustering analysis of scRNA-Seq and scATAC-Seq data, respectively (16, 17). To address batch effects, we applied Harmony’s batch correction algorithm, which effectively normalized the data (18) (Supplemental Figure 1, C and D). We identified 6 scRNA-Seq clusters and 5 scATAC-Seq clusters, which we visualized using Uniform Manifold Approximation and Projection (UMAP) (Figure 1B). These clusters were categorized based on characteristic lineage markers and included T cells (*CD3D*^+^, *CD3E^+^, CD2^+^*), NK cells (*NKG7*^+^, *GNLY^+^*), B cells (*CD79A*^+^, *MS4A1*^+^), myeloid cells (*S100A9*^+^, *LYZ^+^*), cycling (*MKI67*^+^, *STMN1^+^*), and progenitor (*GATA2*^+^, *KIT^+^*) (Figure 1, C and D, and Supplemental Figure 1E). The cycling cluster was annotated as T/NK cells in the cell proliferation cycle due to their high expression of cell cycle–related genes. The progenitor cluster consisted of hematopoietic stem cell–like cells. The distribution of major cell types was consistent across all samples (Supplemental Figure 2, A–L), suggesting that the immune cell subtype composition was largely uniform among the sampled groups.

### Inflammatory immune environment intensifies with GO progression.

To evaluate the severity of GO, we employed a gene set score specific to GD, comparing healthy individuals, patients with GH, and patients with GO. In line with previous reports (1, 3), a stepwise increase in transcriptomic activity was noted across the 3 groups, though chromatin accessibility did not significantly differ between GH and GO groups (Figure 1E). This trend across the healthy, GH, and GO states suggested a progression trajectory for GO. Notably, an increase in GD-related disease scores was observed across nearly all immune cells, both at the transcriptomic and chromatin accessibility levels (Supplemental Figure 3A). Given the heightened inflammatory response associated with the onset of GO (19), we analyzed the expression of inflammation-related genes and the activation of the proinflammatory *IFNG* pathway across the groups. A consistent gradient increase in the expression of inflammatory genes was observed at both the transcriptomic and chromatin accessibility levels (Figure 1E), a trend that consistent across cell types (Supplemental Figure 3, C and E). The scatter plots highlighted a clear correlation between increasing disease severity and inflammation levels (Figure 1F), suggesting a link between the inflammatory state and GO progression. Flow cytometry analysis of peripheral blood samples further confirmed a significant elevation in inflammatory cytokines IL-6, TNF-α, and IFN-γ in patients with GO compared with both healthy and GH groups (Figure 1, G–I), and the majority of cell subpopulations showed this trend (Supplemental Figure 3, C–E).

Furthermore, heatmaps generated from both transcriptomic and chromatin accessibility data revealed that myeloid cells were the potential primary drivers of the heightened inflammatory environment (Supplemental Figure 4A). Examination of the myeloid cell subpopulation (Supplemental Figure 4, B–E) identified that Classical Monocyte4 (*CD14*^+^, *IL1B*^+^) was the primary expressor of the elevated inflammatory environment (Supplemental Figure 4F).

### Overactivation of cytotoxic T effector cells and dysfunction of Treg in GO progression.

**Figure 2:**
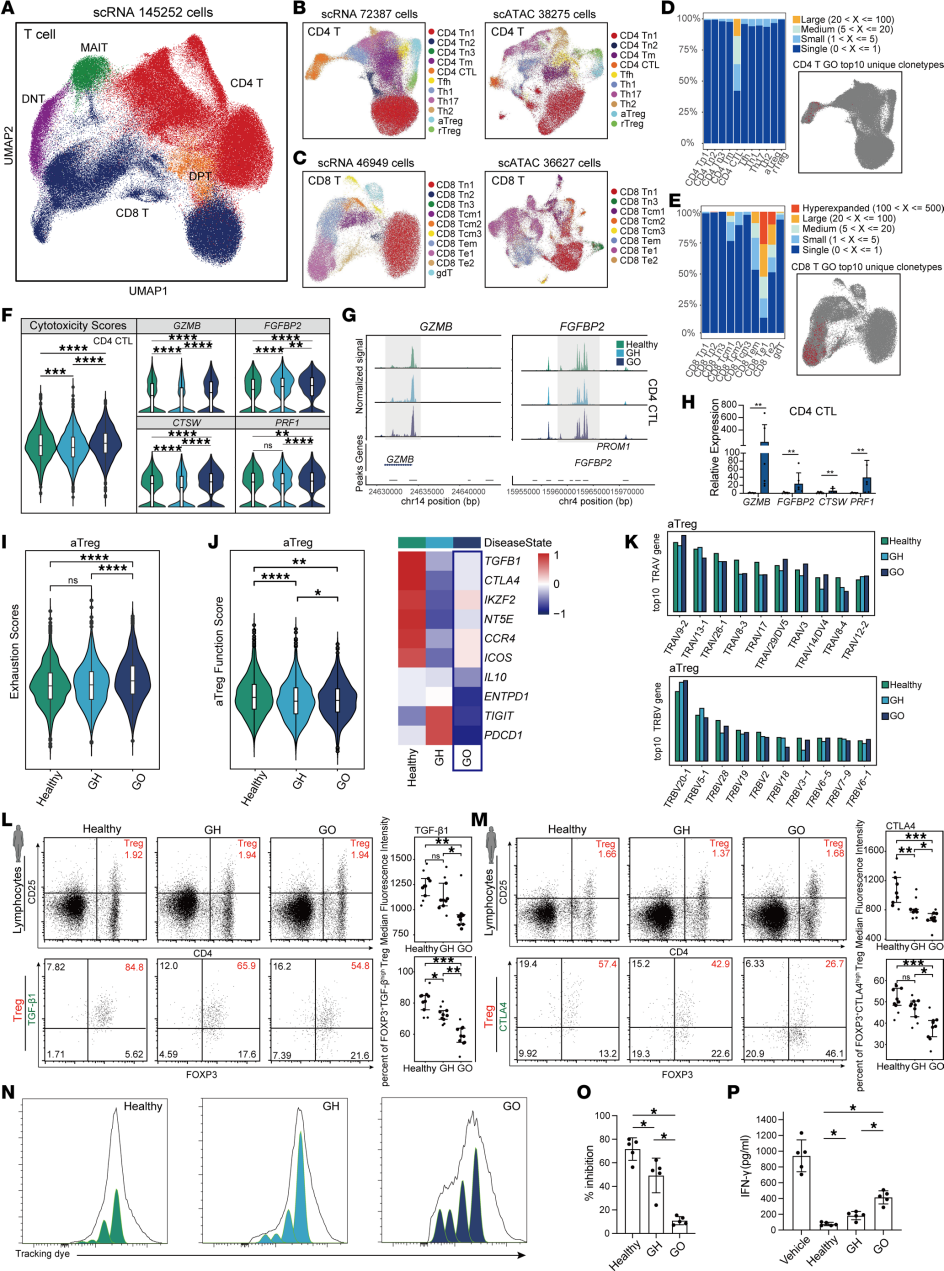
The dynamic transcriptional characteristics of T cell subpopulations during the progression of GO. (**A**) Major T cell subtypes in the scRNA-Seq dataset. (**B** and **C**) All subtypes of CD4 T (**B**) and CD8 T cells (**C**) in both scRNA-Seq and scATAC-Seq datasets. (**D** and **E**) The relative abundance of clonotypes and distribution of the GO top 10 unique clonotype among CD4 T cell subtype (**D**) and CD8 T cell subtype (**E**). (**F**) The expression levels for the cytotoxicity functional genes in CD4 CTL. Data are represented as the median IQR. ****P_adj_ < 0.00001 by Mann-Whitney *U* test. (**G**) Chromatin accessibility peak of *GZMB* and *FGFBP2* in CD4 CTL. (**H**) qPCR showed the gene expression levels of *GZMB*, *FGFBP2*, *CTSW*, and *PRF1* in CD4 CTL (Healthy, *n* = 6; GO, *n* = 6). Data are represented as the median IQR. **P < 0.001 by Mann-Whitney *U* test. (**I** and **J**) The scores of exhaustion score (**I**) and aTreg functional gene score (**J**) in aTreg; heatmap displayed the expression levels of representative functional genes. Data are represented as the median IQR. ****P_adj_ < 0.00001 by Mann-Whitney *U* test. (**K**) The expression levels of the top 10 GO-specific *TRAV* and *TRBV* genes in aTreg. (**L** and **M**) The expression of TGF-β1 (**L**) and CTLA4 (**M**) from 3 groups; dot plots indicate the mean expression intensity and target cell proportions (Healthy, *n* = 9; GH, *n* = 9; GO, *n* = 9). Data are represented as the median IQR. ***P_adj_ < 0.0001 by Mann-Whitney *U* test. *P_adj_ < 0.05, **P_adj_ < 0.001. (**N**–**P**) The proliferation (**N**), inhibition (**O**) and IFN-γ (**P**) production of CD8^+^ T cells co-cultured with Treg (Healthy, *n* = 5; GH, *n* = 5; GO, *n* = 5). Data are represented as the median IQR. *P_adj_ < 0.05 by Mann-Whitney *U* test. The experiments were repeated 3 times.

To explore the T cells’ roles in GO pathogenesis (6, 20), we conducted a comprehensive analysis. T cells were initially categorized into CD4 T cells (*CD3D*^+^, *CD4*^+^), CD8 T cells (*CD3D*^+^, *CD8*^+^), MAIT cells (*TRAV1-2*^+^, *SLC4A10*^+^), DNT cells (*TRDV2*^+^, *TRDC*^+^), and double positive T cells (DPT cells) (*CD4*^+^, *CD8*^+^) based on their transcriptome and chromatin accessibility profiles (Figure 2A and Supplemental Figure 5, A–D). In the scATAC dataset, identifying surface clone antibody genes posed a challenge, so we used alternative markers for MAIT and DNT cells, namely KLRD1 and ROC1 (Supplemental Figure 5C). Further subtyping of CD4 T cells (Figure 2B and Supplemental Figure 5, E and F) and CD8 T cells (Figure 2C and Supplemental Figure 5, G and H) revealed distinct groups. As GO progressed, an increase in cytokine stimulation response scores was observed in most CD4 and CD8 T cell subgroups (Supplemental Figure 5, I and J), indicating an elevated state of reactivity likely driven by the overall inflammatory environment.

Given autoimmune diseases’ nature, involving misidentification and overactivation of self-antigens (21), we identified differential clone types between GO and GH, highlighting unique TCR clone epitopes that emerge as GO progresses, primarily within CD4 CTL and CD8 effector T cell 1 (Te1) cells (Figure 2, D and E). This suggests that CD4 CTL and CD8 Te1 cells may predominantly drive the overactivation during GO progression. Additionally, their cytotoxic capabilities, as evidenced by transcriptomic and chromatin accessibility data, showed a marked upward trend with the disease progression. Key cytotoxic genes, such as *GZMB* and *FGFBP2,* also increased in expression and chromatin accessibility (Figure 2, F and G, and Supplemental Figure 5, K and L). CD4 CTL and *GNLY*^+^ CD8 cells from both healthy and GO donors sorted by FACS confirmed the upregulation of cytotoxic genes including *GZMB*, *FGFBP2*, *CTSW*, and *PRF1*, as assessed by quantitative PCR (qPCR) (Figure 2H and Supplemental Figure 5, M and N). Comparative analysis with the GH group revealed common differential peak motifs in these 2 cell types, such as *ZBTB33* and *KLF15* (Supplemental Figure 6, A and B). No proportional differences between these cell types were observed, indicating that the changes were functional rather than numerical. Furthermore, we identified GO-specific TCRs of CD8 Te1 by examining the expression patterns of top TRAV and TRBV genes (Supplemental Figure 6, C–E).

Differentiation from resting Treg (rTreg) into aTreg is crucial to maintain their suppressive activity during homeostasis and inflammation (22, 23). The pathological development of autoimmune diseases often coincides with dysregulation of Treg function (24), which is consistent with the findings in our study. We identified aTreg (*CD4*^+^, *IL2RA*^+^, *CD44*^hi^) and rTreg (*CD4*^+^, *IL2RA*^+^, *CD44*^lo^) separately. As GO progressed, the exhaustion score of aTreg increased significantly (Figure 2I). Additionally, aTreg exhibited a decline in immunoregulatory function scores and the expression of key immunosuppressive genes (Figure 2J), consistent with flow cytometry results from human donor samples (Figure 2, L and M). To further compare the suppressive function of Treg among the 3 patient groups, isolated Treg and CD8 T cells were cocultured at a 1:1 ratio for 3 days. The inhibition of CD8 T cell proliferation was lowest in the GO patient group (Figure 2, N and O), and the inhibition of IFN-γ production by CD8 T cells was also lowest in the GO patient group (Figure 2P). These results indicate that the immunosuppressive function of aTreg of GO was impaired. The usage of TRAV and TRBV genes among the 3 conditions remained almost unchanged, suggesting that the function of aTreg is in a relatively inert state (Figure 2K).

### The acquisition of CTL transcriptional features by aTreg during GO progression.

**Figure 3:**
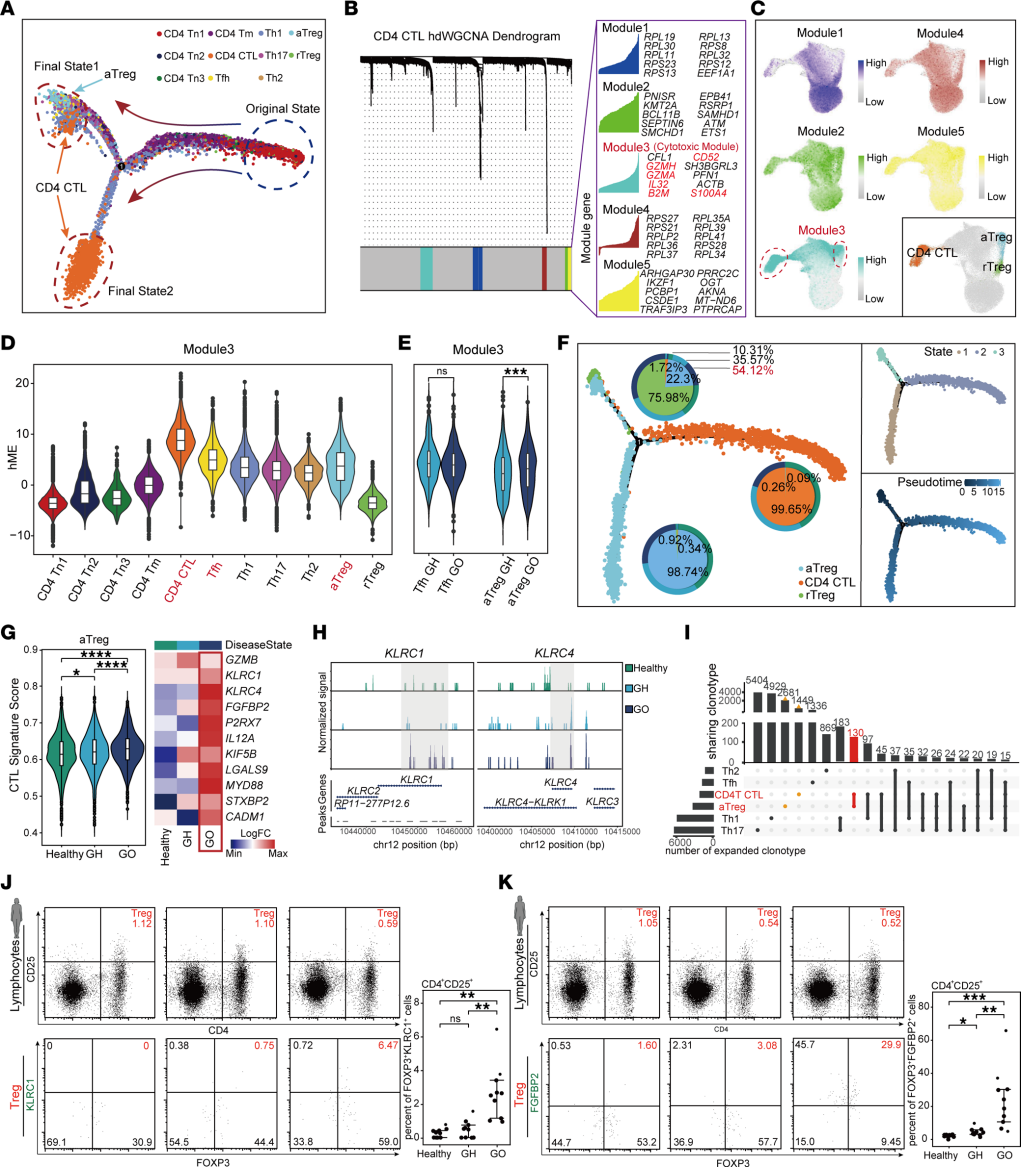
aTreg acquired CTL transcriptional signature under the high-inflammatory conditions of GO. (**A**) Pseudotime analysis of CD4 T cell subpopulations. (**B**) Dendrogram of CD4 CTL hdWGCNA and representative genes for each module. (**C**) UMAP plot showed the expression of different module genes in CD4 T cell subpopulations. (**D**) The expression of module 3 genes in CD4 T cell subpopulations. (**E**) The expression differences of module 3 genes in Tfh and aTreg between GH and GO groups. Data are represented as the median IQR. ***P < 0.0001 by Mann-Whitney *U* test. (**F**) Pseudotime analysis of aTreg, rTreg, and CD4 CTL. Left: Pie chart displayed the proportions of cells in different states over time for Healthy, GH, and GO groups. Right: Distribution of cells in different states along the branching trajectory over time. (**G**) The scores of CD4 CTL transcriptional feature gene set in aTreg from Healthy, GH, and GO groups; heatmap displayed the expression levels of representative CD4 CTL functional genes in aTreg from 3 groups. Data are represented as the median IQR. ****P_adj_ < 0.00001 by Mann-Whitney *U* test. (**H**) Chromatin accessibility plots of *KLRC1* and *KLRC4* genes in aTreg from 3 groups. (**I**) Upset plot of aTreg and CD4 effector T cell clonotype sharing. On left, total number of expanded clones for each subpopulation is displayed. On top, the total number of shared clones is displayed. Incidences of clone sharing between aTreg and CD4 CTL are highlighted. (**J** and **K**) The expression of KLRC1 (**J**) and FGFBP2 (**K**) in peripheral blood CD4^+^CD25^+^FOXP3^+^ Treg from 3 groups; dot plots indicate the target cell proportions (Healthy, *n* = 9; GH, *n* = 9; GO, *n* = 9). Data are represented as the median IQR. *P_adj_ < 0.05, **P_adj_ < 0.001, ***P_adj_ < 0.0001 by Mann-Whitney *U* test.

The role of CD4 subtypes in the development of GO is well documented (7–10). To obtain more dynamic transcriptional characteristics of CD4 T cells relevant to GO, we subjected all CD4 subtypes to pseudotime trajectory analysis. The result revealed a typical developmental pathway: naive CD4 cells differentiated into memory or effector CD4 T cells, leading to 2 distinct states — an immunosuppressive state (state 1) consisting of Treg and a toxic inflammatory state (state 2) comprising CD4 CTL (Figure 3A). Interestingly, CD4 CTL were divided into 2 clusters in the pseudotime trajectory (Figure 3A and Supplemental Figure 7A), which differs from previous pseudotime analysis results (25, 26). Most CD4 CTL were in state 2, while the remaining CD4 CTL were in state 1, which contains other CD4 T cell subsets. This group of CD4 CTL (state 1) may have had distinct transcriptional characteristics from the main CD4 CTL group (state 2) and shared some similarities with other CD4 T cell subsets.

To investigate the transcriptional similarity between CD4 CTL and other CD4 T cell subsets, we applied high-dimensional weighted gene coexpression network analysis (hdWGCNA) (27) to categorize genes in CD4 CTL into modules. Module 3, identified as the Cytotoxic Module, contained numerous inflammatory and cytotoxic genes such as *GZMH* and *GZMA* (Figure 3B). Surprisingly, the cytotoxic module was highly expressed not only in CD4 CTL but also in Tfh and aTreg (Figure 3, C and D). Notably, in the GO group, aTreg showed a significant increase in the expression of genes within the cytotoxic module compared with the GH group, while Tfh did not exhibit such a trend (Figure 3E). Simultaneously, we observed that CD4 CTL in state 1 exhibited higher aTreg transcriptional feature scores (Supplemental Figure 7B). This might suggest that there was an enhanced toxic transcriptional feature in certain aTreg subpopulations during GO progression.

Considering that Treg under inflammatory conditions can convert into other CD4 effector groups such as Th1, Th2, Th17, Tfh, and other proinflammatory phenotypes (14, 24, 28–30), we suspected that there was an aberrant transition of aTreg related to CTL signature within elevated inflammation of GO. We then explored the possibility of CTL phenotypic transition in Treg during GO progression. Pseudotime analyses for rTreg, aTreg, and CD4 CTL revealed 3 states along the pseudotime trajectory (Figure 3F). In the initial state (state 3), a marked proportion of aTreg from the GO group indicated a potential for differentiation during GO development. We confirmed the expression patterns of *FOXP3*, *GZMB*, and *FGFBP2* factors along the pseudotime (Supplemental Figure 7, C and D). Subsequently, the CTL signature score of aTreg as well as the transcription and chromatin accessibility of CD4 CTL characteristic genes, such as *KLRC1* and *KLRC4*, increased with disease progression (Figure 3, G and H). Additionally, there was a considerable degree of aTreg and CD4 CTL clonotype sharing (Figure 3I). Flow cytometry experiments on human GO peripheral blood identified the *KLRC1*^+^ *FGFBP2*^+^ Treg, confirming the conversion of Treg into CD4 CTL during GO progression (Figure 3, J and K), but no tendency toward Th1, Th2, Th17, or Tfh conversion was observed (Supplemental Figure 7, E–H).

To determine if this pathological transition of Treg is common in autoimmune diseases, we analyzed single-cell transcriptome data from various autoimmune diseases, including Behçet’s disease (BD; HRA004778), rheumatoid arthritis (RA; GSE208161), multiple sclerosis (MS; GSE138266), systemic lupus erythematosus (SLE; GSE174188), and primary Sjögren’s syndrome (pSS; GSE157278). After standard data processing, we obtained CD4 T cell subtype atlases for each disease (Supplemental Figure 8, A–E). We noted a common increase in the inflammatory environment in these diseases, with a significant reduction in aTreg functionality and an increase in CTL signature scores, except in RA. This suggested that, in the peripheral blood of autoimmune disease patients, aTreg might exhibit decreased function and acquire CTL signature, influencing disease progression within a generalized inflammatory environment.

### The potential regulation of YY1 on the dysfunction and cytotoxic transition of antigen-specific Treg under high inflammatory condition.

**Figure 4:**
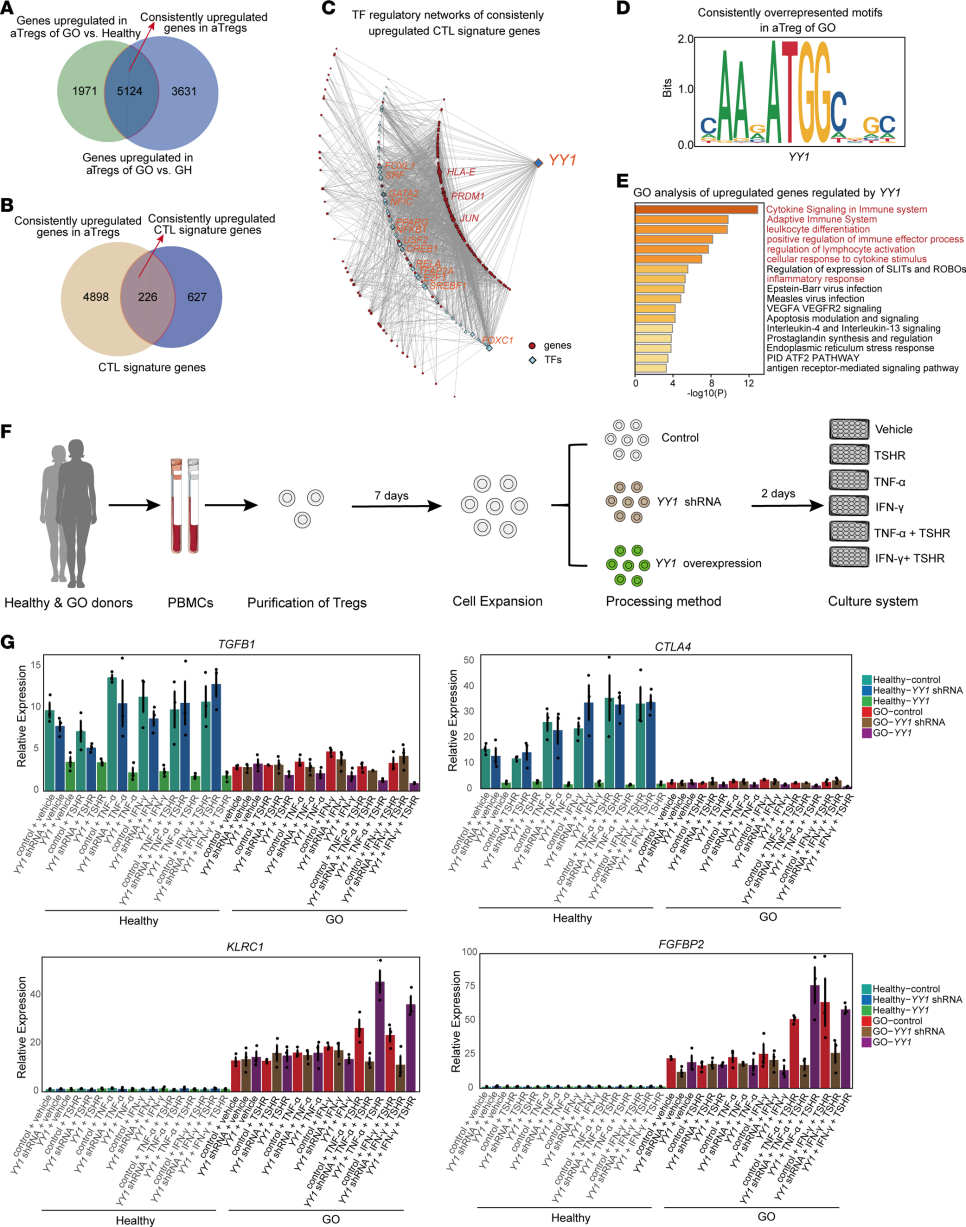
*YY1* promoted the cytotoxic transition of autoreactive Treg upon secondary antigen stimulation in an inflammation-dependent manner. (**A**) Venn diagram illustrated the number of consistently upregulated DEGs in aTreg of GO group compared with the GH group and healthy group. (**B**) Venn diagram displayed the overlap between the consistently upregulated DEGs in aTreg of GO group and the CTL signature gene set. (**C**) The transcription factor regulatory network of aTreg consistently upregulated CTL signature genes predicted based on JASPAR. (**D**) TF motif enrichment analysis of consistently overrepresented sequences in GO. (**E**) GO analysis of consistently upregulated genes regulated by *YY1*. (**F**) Experimental model diagram of the toxic shift of Treg in vitro: Treg were isolated from healthy and GO donors, expanded in culture for 7 days, followed by LV-*YY1* shRNA knockdown or LV-*YY1* overexpression. After 2 days, these cells were treated with TSHR recombinant protein and/or proinflammatory cytokines (TNF-α, IFN-γ) for 48 days. (**G**) qPCR histogram showed the expression levels of 4 specific genes about the results of Treg cytotoxic transition under different treatment conditions. All intergroup *P* values can be found in Supplemental Table 5. Each group consists of 3 independent samples (each *n* = 3). Data are represented as the median IQR. All experiments were repeated 3 times.

To investigate the potential mechanism underlying Treg phenotypic transition, we first identified a gene set that was consistently upregulated in aTreg from healthy, GH, to GO conditions (Figure 4A) and intersected it with the characteristic gene set of CD4 CTL (Figure 4B). This analysis yielded a set of 226 genes, including *FGFBP2* and *KLRC4*. Subsequently, we predicted key transcription factors regulating these genes using the JASPAR TF binding site database. Additionally, we conducted motif enrichment analysis on disease-differential accessible peaks in scATAC-Seq data to identify transcription factors that may persistently regulate the upregulated peaks. Finally, we identified a master regulatory transcription factor, yin yang 1 (*YY1*) (Figure 4, C and D). Furthermore, GO analysis of *YY1*-regulated genes revealed enrichment in pathways related to “Cytokine Signaling in Immune System”, “Adaptive Immune System”, “Leukocyte Differentiation”, “Positive Regulation of Immune Effector Process”, “Regulation of Lymphocyte Activation”, “Cellular Response to Cytokine Stimulus”, and “Inflammatory Response” (Figure 4E). Previous studies have reported that *YY1* is upregulated in Treg under inflammatory conditions and negatively regulates Treg function and *FOXP3* activity (31, 32). Based on these findings, we hypothesized that *YY1* may alter the normal function and phenotype of Treg in the high-inflammatory environment of GO.

To further validate the potential regulatory role of *YY1*, we sorted *CD4*^+^*CD25*^+^ Treg from PBMCs of healthy donors and patients with GO. After in vitro cell expansion, we knocked down or overexpressed *YY1* in Treg (Figure 4F). The cells were then treated with proinflammatory cytokines (TNF-α and IFN-γ) and/or GO-specific antigens, TSHR protein (Figure 4F). Notably, Treg (sorted from PBMCs of patients with GO) treated with proinflammatory cytokines and TSHR showed significant downregulation of the functional genes *CTLA4* and *TGFB1* and upregulation of the toxicity factors *FGFBP2* and *KLRC1* (Figure 4G). This indicates the transition in Treg occured only in the inflammatory environment when the TSHR antigen was present (Figure 4G). In other words, it required the execution of a secondary immune response by Treg with immune memory. Meanwhile, this phenomenon became more pronounced upon overexpression of the *YY1* gene but disappeared after *YY1* KO (Figure 4G). These findings validate the autoreactivity, antigen-experienced Treg isolated from patients with GO in response to autoantigens in an inflammatory environment, and supporte the bioinformatics interpretation that *YY1* could regulate aberrant function decline and phenotypic transition of Treg.

### Corticosteroid therapy mitigated inflammation and altered immune cell profiles in GO.

**Figure 5:**
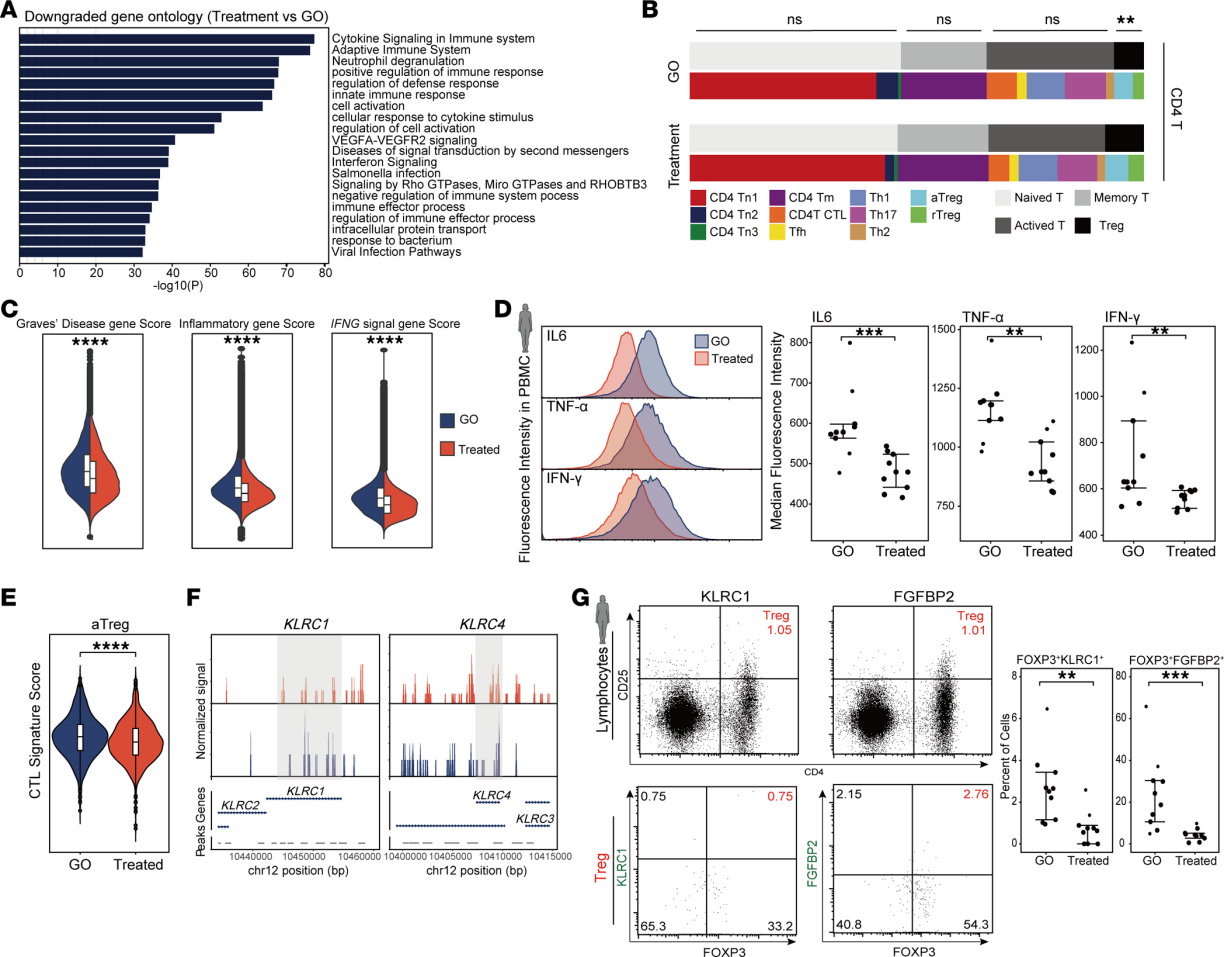
Cytotoxic phenotype of Treg was alleviated as inflammation decreased after corticosteroid therapy. (**A**) Bar chart showed the GO functional enrichment of downregulated DEGs in the corticosteroid-treated group compared with the GO group. (**B**) The cell proportions of CD4 T cell subpopulations within the GO and Treated groups. Data are represented as the median IQR. **P < 0.001 by Mann-Whitney *U* test. (**C**) The expression and activity scores of Graves’ disease, inflammatory, and *IFNG* signal gene sets between the GO and Treated groups. Data are represented as the median IQR. ****P < 0.00001 by Mann-Whitney *U* test. (**D**) Flow density plots and dot plots showed the expression intensity of IL-6, TNF-α, and IFN-γ in peripheral blood PBMCs from the GO and Treated groups (GO, *n* = 9; Treated, *n* = 9). Data are represented as the median IQR. ***P < 0.0001 by Mann-Whitney *U* test. **P_adj_ < 0.001. (**E**) Violin plots show the CTL signature score of aTreg between the GO and Treated groups. Data are represented as the median IQR. ****P < 0.00001 by Mann-Whitney *U* test. (**F**) Chromatin accessibility plots for *KLRC1* and *KLRC4* genes in aTreg between the GO and Treated groups. (**G**) Flow cytometry plots displayed the expression of KLRC1 and FGFBP2 in peripheral blood CD4^+^CD25^+^FOXP3^+^ Treg in Treated groups; dot plots indicating the target cell proportions (GO, *n* = 9; Treated, *n* = 9). **P_adj_ < 0.001. Data are represented as the median IQR. ***P < 0.00001 by Mann-Whitney U test.

We evaluated the effects of corticosteroid therapy on GO by including a cohort of 11 patients with inactive GO (CAS < 3) who received methylprednisolone pulse therapy. Utilizing the same sequencing and analytical approaches, we generated a single-cell atlas for these treated patients with GO (Supplemental Figure 9, A–C). Posttreatment gene expression analysis indicated a substantial reduction in inflammatory and immune response gene pathways (Figure 5A). There was also a notable decrease in the enrichment scores of genes related to GD, general inflammation, and the *IFNG* proinflammatory signaling pathway (Figure 5C and Supplemental Figure 9D). Flow cytometry analysis also confirmed that the fluorescence intensity of IL-6, TNF-α, and IFN-γ in peripheral blood of the treated group was lower compared with the GO group, indicating a reduction in inflammation levels in the peripheral blood following corticosteroid therapy (Figure 5D).

We then assessed the effect of corticosteroid therapy on T cell subsets. There was a marked increase in the proportion of Treg (Figure 5B). The cytotoxic scores of CD4 CTL and CD8 Te1 cells were also diminished (Supplemental Figure 9E), along with decreased chromatin accessibility for cytotoxic genes such as *FGFBP2* and *GZMB* (Supplemental Figure 9F). Within the Treg subsets, the heatmap shows that part of a functional-related gene of aTreg recovered (Supplemental Figure 9G), but there was no notable change in immunoregulatory function scores or flow cytometry (Supplemental Figure 9, H and I). Additionally, in vitro suppression assays show that, compared with the GO group, Treg from the treated group did not exhibit notable changes in the inhibition of CD8 T cell proliferation or in the suppression of IFN-γ production by CD8 T cells (Supplemental Figure 9, J–L). Notably, the CTL signature score of aTreg markedly decreased (Figure 5E), as did the chromatin accessibility of cytotoxic genes like *KLRC1* and *KLRC4* (Figure 5F), which were corroborated by flow cytometry (Figure 5G).

The analysis of TCR clonotypes revealed a substantial reduction in GO-specific clonotypes within CD4 CTL and a modest decrease in CD8 Te1 cells after treatment (Supplemental Figure 9M). The diversity of the amino acid repertoire in the CDR3 region of CD4 CTL also showed a substantial decrease (Supplemental Figure 9N).

In conclusion, methylprednisolone pulse therapy effectively ameliorated the inflammatory state in GO, reversed the toxic transcriptional signature in aTreg, and diminished the presence of GO-specific T cell clonotypes. These findings provide insights into the cellular and molecular changes associated with clinical improvement in GO.

### Pathogenic infiltration into orbital tissues of cytotoxic Treg in GO mouse model.

We developed a GH/GO mouse model in Balb/c mice by administering an intramuscular injection of adeno-associated virus–TSHR (AAV-TSHR) into the anterior tibial muscle (Supplemental Figure 10A) (33, 34). Animals were randomly divided into 2 groups, with the immunization administered every 4 weeks. One group underwent successful GH model assessment after 3 injections, while the other group was assessed for successful GO modeling after 6 injections. In the GH group, mice with elevated blood T4 levels, increased TSHR, decreased TSH levels, and negative MRI results (no substantial increase in orbital soft tissue volume) were considered successful GH models. In the GO group, mice with elevated blood T4 levels, increased TSHR, decreased TSH levels, and MRI evidence of increased orbital soft tissue volume were considered successful GO models. Successfully modeled mice were used for subsequent experiments (Supplemental Figure 10, B and C). Mirroring our human data, the GO mouse model also displayed elevated levels of inflammatory cytokines such as IL-6, TNF-α, and IFN-γ in the peripheral blood (Supplemental Figure 10, D–F), a reduction of Treg-associated functional proteins TGF-β and CTLA4 (Supplemental Figure 10, G and H), and an increase of cytotoxic proteins KLRC1 and GZMB in Treg (mice do not have the *FGFBP2* gene) (Supplemental Figure 10, I and J). These findings indicate a high-inflammatory state and the pathological transition of Treg to CD4 CTL in the GO mouse model, akin to the human condition (Supplemental Figure 10K). Following treatment with methylprednisolone via i.p. injection (35), we observed results in the mouse model that were consistent with those in human samples (Supplemental Figure 10, L and M).

**Figure 6:**
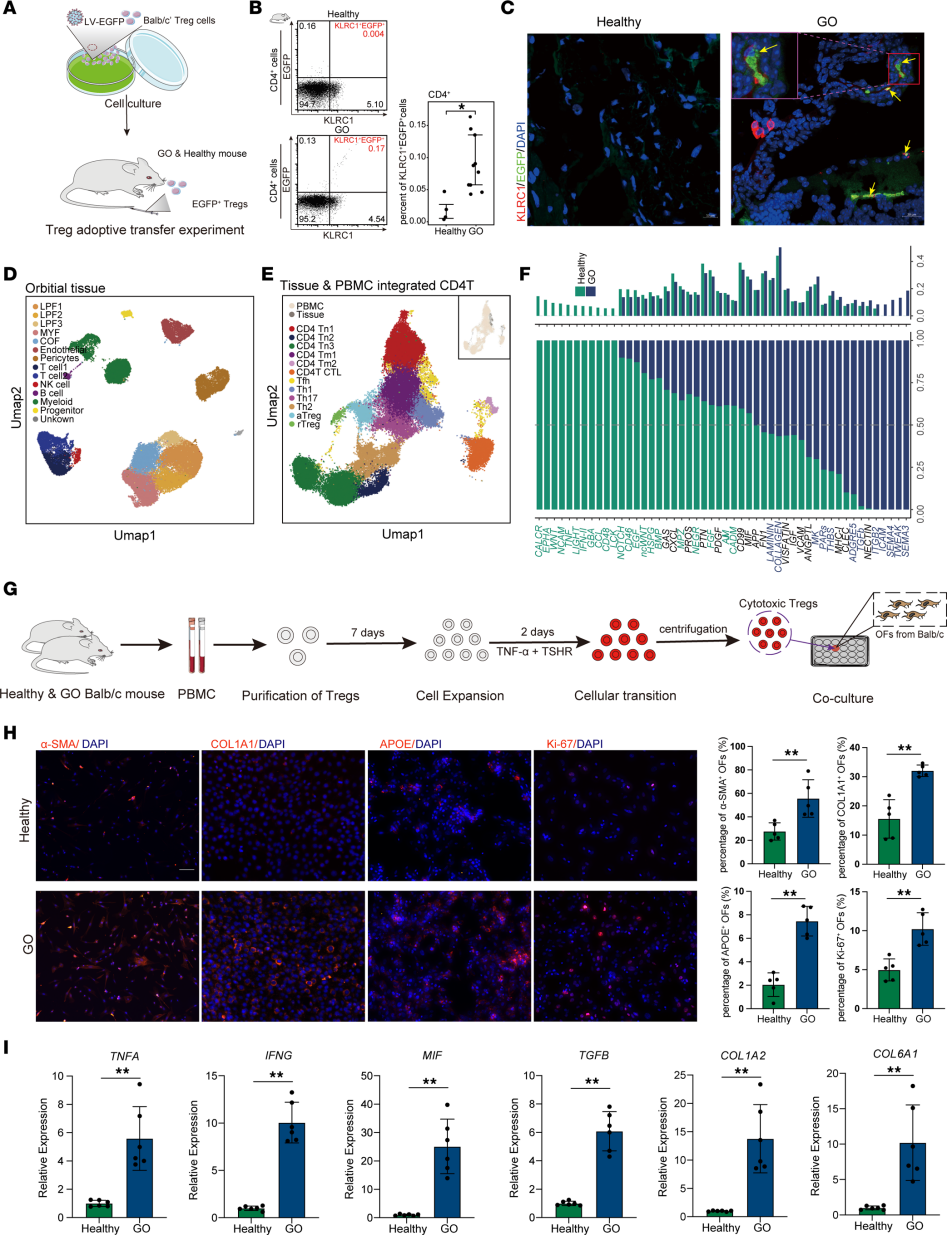
Pathogenic role of Treg cytotoxic transition to the orbital lesions of GO. (**A**) Schematic diagram of the EGFP labeled autoreactive Treg adoptive transfer experiment. (**B**) The proportion of EGFP^+^KLRC1^+^ cells within CD4^+^ cells in the peripheral blood of Healthy and GO mice after adoptive transfer experiment, representing the cytotoxic transition of Treg in vivo (Healthy, *n* = 3; GO, *n* = 9). Data are represented as the median IQR. *P < 0.05 by Mann-Whitney *U* test. (**C**) Immunofluorescence staining demonstrated the presence of KLRC1^+^EGFP^+^ cells in the orbital tissues of GO model mice. (**D** and **E**) The major subtypes of orbital tissues (**D**) and integrated CD4 T cells from PBMCs and tissues (**E**) based on scRNA-Seq data of Healthy donors and patients with GO; the upper right UMAP plot illustrates the batch effects. (**F**) The ligand-receptor interactions involving CD4 CTLs significantly altered in GO among 3 LPF subgroups, MYF, and COF. lipofibroblast, LPF; myofibroblast, MYF; conventional orbital fibroblast, COF. Red represents inflammation-regulating ligand-receptor interactions, while green indicates ligand-receptor interactions related to extracellular matrix remodeling. (**G**) Experimental model of the pathogenic effect of Treg cytotoxic transformation on localized lesions in the orbit. (**H**) Immunofluorescence showed the expression of specific proteins of OF cells in the coculture system of healthy control and GO model mouse groups; the histograms represent the quantitative statistics of these proteins. Data are represented as the median IQR. **P < 0.001 by Mann-Whitney *U* test. (**I**) qPCR histogram showed the expression of specific genes in cells of the coculture system. Each group consists of 3 independent samples (each *n* = 6). Data are represented as the median IQR. **P < 0.001 by Mann-Whitney *U* test. All experiments were repeated 3 times.

We then investigated whether CD4 CTLs, transformed from Treg in the peripheral blood, could infiltrate the orbital tissues (4). CD4 T cells were isolated from the spleens of adult Balb/c mice and polarized in vitro with TGF-β, resulting in 96.7% of cells differentiating into Treg (Figure 6A and Supplemental Figure 11A). These Treg were transduced with a lentivirus encoding EGFP, sorted to high purity using Treg magnetic beads, and injected into the GO mouse model via the tail vein (Figure 6A). Flow cytometry analysis detected a considerable population of *KLRC1*^+^*EGFP*^+^ CD4 T cells in the blood of GO mice, indicating partial cytotoxic transition of the EGFP-tagged Treg (Figure 6B). Immunofluorescence staining further revealed *KLRC1*^+^*EGFP*^+^ double-positive cells within the orbital tissues (Figure 6C). These results suggest that these converted CD4 CTLs were capable of infiltrating the orbital soft tissues and may contribute to the pathogenesis of GO.

### Promotion of cytotoxic Treg on orbital inflammation and OFs stromal remodeling in GO.

Our investigation into the role of CD4 CTL in GO revealed their affect on the progression of orbital lesions. We analyzed a single-cell dataset of orbital tissues from patients with GO (GSA: HRA000870) (5). After stringent quality control and annotation, we focused on identifying fibroblast subgroups due to their relevance in GO pathology. These subgroups included various lipofibroblast (LPF) and MYF subtypes, as well as conventional OFs (COF) (Figure 6D and Supplemental Figure 11B). Compared with healthy controls, all fibroblast subgroups in patients with GO exhibited increased expression of adipogenesis and ECM genes (Supplemental Figure 11D), highlighting their contribution to the pathological changes in orbital tissues (1). We then integrated PBMC data from patients with GO with T cell data from orbital tissues (Figure 6E and Supplemental Figure 11C). To understand the role of these infiltrating CD4 CTLs, we analyzed cell communication signals between CD4 CTLs and fibrosis-related cell subgroups. The analysis revealed upregulated signaling pathways in CD4 CTLs, including those related to LAMININ, COLLAGEN, MK, and ICAM (Figure 6F and Supplemental Figure 11F), which are known to be associated with fibrosis (36–39). This suggested that CD4 CTLs may contribute to fibrosis and stromal remodeling by interacting with fibroblast subgroups. Further examination of specific receptors on CD4 CTLs showed that in GO, these cells could influence inflammation-regulating pathways associated with various fibroblast subgroups and upregulate receptors related to stromal remodeling, such as *TGFB*, *AREG*, and *COL1A1* (Supplemental Figure 11E) (40). This suggested a profound effect on the inflammatory environment and stromal remodeling processes in the orbit of patients with GO, potentially driving the local progression of the disease.

To investigate whether the Treg with CTL phenotype was present as part of orbital pathogenic CD4 CTL, we isolated autoreactive Treg from GO mouse model (Figure 6G). We found that, consistent with the Treg of patients with GO, antigen-experienced Treg of the GO mouse model showed toxic phenotypic transition after treatment with TSHR and proinflammatory factors (Supplemental Figure 11G). Next, we separated the cytotoxic Treg from the culture system and cocultured them with the OFs from healthy mice (Figure 6G). Immunofluorescence showed that the expressions of OFs proliferation-related protein Ki-67, fibrosis-related protein α-SMA, COL1A1, and adipogenic-associated protein APOE were markedly increased when cocultured with cytotoxic Treg (Figure 6H). qPCR results show an increase in the expression levels of *TGFB*, *COL1A2*, and *COL6A1* genes, which are related to matrix regulation–related pathways in OFs (Figure 6I). Additionally, qPCR results of all cells exhibited an increase in inflammatory cytokines *TNFA*, *IFNG*, and *MIF* (Figure 6I). These results validate our pathway analysis (Figure 6F), indicating the promotive role of the cytotoxic Treg in the inflammatory environment and fibrotic processes of orbital local leisions.

In conclusion, our findings suggest that, in the context of a high-inflammatory blood environment, Treg can acquire cytotoxic phenotype. These cells, through their interactions with disease-related OFs, can influence the local orbital inflammatory environment, promoting stromal remodeling and fibrosis characteristic of GO.

## Discussion

GD is a common autoimmune condition that often progresses to GO, leading to irreversible pathological changes in the orbital tissues and resulting in ocular manifestations and functional impairments (1, 3). Effective identification strategies for individuals in the early stages of GO may be crucial for preventing disease progression. However, prior research has largely focused on the transcriptomic shifts in GO at advanced stages, when symptoms are already evident (4, 5). Our study addresses this gap by applying single-cell multiomics to map pivotal gene expression changes, antigenic clonal epitopes, and chromatin accessibility throughout the disease’s progression. Our study concluded that, in the high-inflammatory environment, aberrant regulation by *YY1* led to the impairment of immunosuppressive function and cytotoxic phenotypic transition of GO-autoreactive Treg. These cells infiltrated orbital lesion tissues and promoted pathogenic fibrosis in OFs, contributing to the progression of GO.

The inflammatory milieu within orbital tissues is a well-documented driver of GO pathogenesis (4, 5, 41–43). Active patients with GO present with heightened inflammatory responses in ocular regions, marked by eyelid swelling and redness (15). Moreover, initial inflammatory levels are deemed a predictive factor for GO development in individuals with GD (1–3, 43, 44). Our findings supported this, demonstrating a stepwise intensification of GD severity transitioning into GO, paralleled by a marked increase in the inflammatory profile of the systemic immune milieu. This trend was reflected across all 6 principal immune cell types and their subsets, indicating a immune dysregulation in the bloodstream during GO progression. We proposed that a systemic high-inflammatory state may be a characteristic pathophysiological hallmark associated with the onset and progression of orbital lesions in GO.

T cells play a crucial role in the pathogenesis of GO (6, 20). Within this inflammatory milieu, we explored the epigenetic and transcriptional shifts in T cell subsets. As the disease escalates, all CD4 and CD8 T cell subsets exhibited a marked rise in cytokine response sensitivity, heightening their reactivity. CD4 CTL and CD8 Te1, known for their cytotoxic roles in autoimmune conditions like ulcerative colitis, were implicated in local lesion exacerbation by upregulating inflammatory mediators (45, 46). Our analysis indicated that both subsets showed enhanced cytotoxicity, with sustained increases in expression and chromatin accessibility of key cytotoxic genes such as *GZMB*, *FGFBP2*, *CTSW*, *PRF1*, which are crucial in other autoimmune disorders (47–49). While the proinflammatory effect of CD4 CTL in GO has been previously discussed (4, 33), we focused on the disease-specific cytotoxic transcriptional traits of this subset in the bloodstream, alongside chromatin accessibility shifts linked to cytotoxic function changes. Furthermore, we identified TFs and their motifs tied to disease progression. Beyond functional disparities, we noted that these cell types closely correlate with GO-specific T cell antigenic epitopes, pinpointing unique antigen clones like *TRAV1-2* and *TRBV9*, which could pave the way for novel therapeutic strategies in GO management.

Treg play a pivotal role in maintaining self-tolerance and mediating immune suppression. Despite the well-documented regulatory functions of Treg in various autoimmune conditions (50, 51), their specific roles in GO remain underexplored. Intriguingly, our study revealed that the expression of cytotoxic module genes in aTreg increased during the disease’s progression. Given the known propensity of Treg for partial instability and transdifferentiation (24, 29, 30), this observation suggested a trend of toxic phenotypic transition of Treg in the highly inflammatory milieu characteristic of GO progression. Treg can exhibit unstable phenotypes in high-inflammatory conditions, predisposing them to differentiate into various effector CD4 subpopulations within an inflammatory setting, thereby potentially exacerbating immune-mediated diseases (24, 30, 52). Previous studies, such as those by Komatsu et al. (24) and Jiang et al. (30) have shown that, in RA and SLE, the unstable conversion of Treg to Th17 cells could aggravate disease severity. Similarly, Zhou’s work highlighted the pathological transition of Treg to memory effector T cells in the context of autoimmune diabetes (52). However, these studies have yet to document the abnormal transition of Treg to a cytotoxic phenotype.

Besides, more aTreg in patients with GO entered the initiation state of differentiation, suggesting a potential shift toward effector CTL characteristics. Moreover, aTreg displayed an increased signature of CTL markers and enhanced transcriptional activity and chromatin accessibility of cytotoxic genes such as *KLRC1* and *KLRC4*. We identified an increased population of *FOXP3*^+^*KLRC1*/*FGFBP2*^+^ Treg (*CD4*^+^*CD25*^+^) in the GO group, both in human blood samples and mouse models, providing robust evidence for our findings. Further analysis across other autoimmune diseases revealed a similar cytotoxic transition of Treg, suggesting that this phenomenon might be a common feature across autoimmune conditions.

Previous studies have shown that, under inflammatory conditions, *YY1* increases in Treg, disrupting the *Foxp3*-dependent target gene expression by physically interacting with *Foxp3* and directly binding to *Foxp3* target genes, thereby inhibiting the differentiation and function of Treg (31, 32). In our study, autoreactive Treg exhibited reduced function and underwent a cytotoxic transition upon secondary stimulation of TSHR under inflammatory conditions, which is abnormally regulated by *YY1*. Therefore, we speculated that *YY1* may not only have influenced the normal function of Treg but also potentially induced a transition of the unstable phenotype of Treg by downregulating the activity of *FOXP3* (24, 31, 53).

Notably, we directly observed the cytotoxic Treg carrying *EGFP* in the peripheral blood of the GO mouse model, with these cells infiltrating orbital tissues, indicating their direct involvement in lesion progression. Additionally, through coculturing cytotoxic Treg with OFs, we elucidated that cytotoxic Treg upregulated local inflammation levels and participated in disease-associated cellular fibrosis and stromal remodeling. In this highly inflammatory context, the Treg functionality declined and phenotype transformed, which is considered a key mechanism in the pathogenesis of various immune diseases (24, 30, 52).

The primary antiinflammatory treatment for GO, as per current clinical guidelines, is high-dose corticosteroid therapy (15). Our findings suggest that corticosteroid pulse therapy can reverse several cellular and molecular phenotypes associated with GO progression, effectively mitigating the inflammatory milieu, reducing cytotoxic functions of CD4 CTL and CD8 Te1, and diminishing the GO-specific clonal phenotype. Crucially, it appeared to prevent the cytotoxic transition of aTreg. These insights not only shed light on the cellular and molecular dynamics after GO treatment but also reinforce the link between cellular transcriptional shifts and the active phenotype of GO. However, the restoration of Treg functionality after treatment remained elusive, which might have been one of the reasons why the disease phenotype did not reverse and even relapsed after methylprednisolone pulse therapy. This suggests that greater attention should have been given to addressing the Treg functional abnormalities in patients with GO. Additionally, with patients with GH having a less inflammatory background than patients with GO, the decline in Treg function was less severe. We speculated that early control of the inflammatory environment might have reduced the irreversible decline in TREG function in a timely manner, emphasizing the potential preventive value of corticosteroid therapy in high-risk patients with GH to prevent GO development (3, 15).

Although this study offers a comprehensive overview of systemic immune state dynamics during GO disease progression, several limitations should be acknowledged. Firstly, the sample size and diversity are limited, and the genetic, environmental, and immunological complexities may affect the generalizability of the results. Secondly, the uneven sex distribution within the patient population may amplify sex-related cellular and molecular differences. Future studies should include a more diverse patient population covering different stages of the disease to better capture genetic variations across various phases of the progression. Expanding the research to larger cohorts and incorporating genome-wide association studies (GWAS) and proteomics analyses would further deepen our understanding and clarify the key mechanisms underlying the progression of GD to GO. Additionally, the molecular mechanisms by which *YY1* regulates Treg identity transition in high-inflammatory environments remain unclear. Further investigation into these interactions will be essential for refining clinical interventions and improving disease management.

In conclusion, our single-cell multi-omics atlas offers a detailed characterization of peripheral blood immune cells throughout GO progression, enhancing our understanding of the disease’s pathogenic mechanisms. We believe that this study not only sheds light on the systemic immune responses influencing local orbital lesions but also opens avenues for the development of novel, targeted therapeutic interventions for GO. The phenomenon reported in this study about cytotoxic transition of functionally impaired aTreg within a high-inflammatory environment, also noted in other autoimmune diseases, provided insights into the mechanisms of self-tolerance breakdown in autoimmune conditions. We assume that such cytotoxic aTreg may serve as novel biomarkers for autoimmune diseases and potentially become new targets for targeted therapy. Moreover, despite growing hope that Treg-based therapies will offer a cure to autoimmune diseases, our study highlights the importance of considering the inflammatory environment within patients. Further investigation is needed to determine whether exogenous Treg may undergo functional impairment and cytotoxic conversion under specific conditions, which could lead to reduced or even lost therapeutic efficacy.

## Methods

### Sex as a biological variable.

In our human study, our sample was randomly selected, including both male and female participants. Similar findings are reported for both sexes, and sex was not considered a biological variable. In our animal experiments, following the method described by other researchers, female BALB/c mice were recommended as the classical model for GO (35), so we used only female mice for experiments and intergroup comparisons. In this context, sex was not considered a biological variable.

### Patients.

The study included a total of 41 PBMC samples, and characteristics such as sample sex, age, and disease status are detailed in Supplemental Table 1. Among these, 10 samples were from healthy volunteers, 10 samples were from patients with GH without ocular symptoms, 10 samples were from newly diagnosed patients with GO, and 11 samples were from patients with GO revisited after corticosteroid therapy. Exclusion criteria for samples included the presence of other acute or chronic inflammatory diseases and various comorbidities such as cancer, immunodeficiency, diabetes, and other autoimmune diseases. There were no statistically significant differences in the distribution of age and sex between the groups (Supplemental Table 1). Patients were scored based on the CAS (15), with newly diagnosed patients using the 7-item CAS (CAS ≥ 3) and follow-up patients after treatment using the 10-item CAS (CAS < 3). According to Li’s description (5), the orbital tissue single-cell database (GSA: HRA000870; https://ngdc.cncb.ac.cn/gsa-human/browse/HRA000870) was obtained from healthy cohorts and patients with GO who underwent orbital decompression surgery (CAS ≥ 3).

### Mouse models.

Four- to 8-week-old Balb/c female mice purchased from GemPharmatech Co. Ltd. Mice with abnormal manifestations such as eye trauma or tumors were excluded. All baseline information of mice, including housing conditions, experimental procedures, and sample collection methods, were the same between mice.

### PBMC suspension preparation.

We collected peripheral venous blood samples from patients and processed them using Ficoll-Hypaque density solution and heparin. After centrifugation for 30 minutes, the PBMC layer was extracted. PBMCs were stained with Trypan Blue (Biosharp, BL627A) after dilution with PBS (MilliporeSigma, P2272) to assess viability and quantity. Each PBMC sample was split for scRNA-Seq and scTCR-Seq, and the other for scATAC-Seq, maintaining cell viability above 90% on ice.

### scRNA-Seq library preparation.

Single-cell suspensions from the samples were prepared using the Chromium Single Cell 5′ Kit v2 (10X Genomics, PN-1000263) and Chromium Single Cell V(D)J Reagent kits (10X Genomics, PN-1000252 [TCR]), following the manufacturer’s instructions. Libraries were constructed for barcoding and sequencing on the 10X Genomics platform using Chromium Single Cell Library, Gel Beads, and Multi Reagent Kits. Sequencing was performed on an Illumina NovaSeq6000 with PE150 reads, and quality was assessed using FastQC. The raw data were processed and aligned to the GRCh38 reference.

### Processing workflow of scRNA-Seq data.

Using Seurat V4, we filtered low-quality cells, excluding those with > 20% mitochondrial gene expression, < 200 detected genes, or > 4,000 genes. Clusters expressing HBB, HBA1, PPBP, and PF4 were also removed. We normalized and scaled the top 2,000 variable genes with FindVariableFeatures and performed PCA (30 components). Batch effects were corrected using Harmony, and k-nearest neighbors (KNN) clustering (resolution = 1.0) was performed, followed by UMAP analysis. We applied DoubletFinder to eliminate potential doublets, annotating clusters based on feature gene expression (Supplemental Table 2). Public scRNA-Seq data underwent the same pipeline.

### TCR V(D)J immune complex sequencing and analysis.

As previously described, full-length TCR V(D)J segments were obtained by PCR amplification from 5′ libraries using a Chromium Single-cell V(D)J Enrichment kit. Demultiplexing, gene quantification, and TCR clonotype assignment were performed using the Cell Ranger (v.6.0.0) V(D)J pipeline with GRCh38 as the reference genome. The V(D)J immune repertoire was analyzed using scRepertoire with its tutorials.

### scATAC-Seq library preparation.

Single-cell nuclei were isolated, washed, and counted as previously described (54). The isolated nuclei were immediately used to establish 10x single-cell ATAC libraries at Berry Genomics Co. Ltd. Sample processing, library preparation, and sequencing were performed according to the 10x Chromium guidelines. Raw sequencing data were demultiplexed into fastq format using CellRanger-atac-mkfastq (10X Genomics, v.1.0.0). Subsequently, scATAC-Seq reads were aligned to the GRCh38 reference genome and quantified using the CellRanger-atac count function (10X Genomics, v.1.0.0).

### Processing workflow of scATAC-Seq data.

Data processing followed Signac (v.1.12.0) guidelines (17). Peak sets were converted to genomic ranges using the makeGRangesFromDataFrame function, and a unified peak set was generated with the reduce function, filtering out poor-quality peaks (length < 10,000 or > 20). Cells with low counts (<1,000) were excluded. Fragment objects were read, and peak quantification was performed using FeatureMatrix and CreateChromatinAssay. Selected cells had expression levels of 3,000 to 30,000; > 15 ATAC-Seq fragments per peak; and TSS enrichment score < 4. Standardization, feature selection, dimensionality reduction, and batch effect removal were executed with RunTFIDF, FindTopFeatures, RunSVD, and Harmony (18). UMAP and gene activity expression matrix creation were conducted for cell cluster analysis (Supplemental Table 2).

### Integrating with scRNA-Seq data.

We integrated scATAC-Seq data with scRNA-Seq data using the FindTransferAnchors function. The prebuilt Seurat object from scRNA-Seq served as the reference for integration.

### Analysis of gene expression levels and gene activity.

After constructing scRNA-Seq and scATAC-Seq matrix objects, we used the DimPlot function and heatmap format to visualize gene expression levels and gene activity (chromatin accessibility).

### Gene set score analysis.

All gene set scores (including expression scores and gene activity scores) were calculated using the Seurat function AddModuleScore. The gene sets were sourced from the Gene Ontology (GO) website. A summary of the gene set information is provided in Supplemental Table 3.

### Differential gene analysis.

Differential gene expression analysis utilized Seurat’s FindMarkers function with the MAST test, selecting DEGs based on adjusted |logFC| > 0.25 and adjusted *P* < 0.05. The Top 50 marker genes for clustered cells are in Supplemental Table 2. The TF regulatory network was analyzed using NetworkAnalyst with the JASPAR TF-gene interaction database.

### Peak calling analysis.

The CallPeaks function in Signac was employed for peak calling. The FeatureMatrix function was utilized to quantify the counts for each peak, and the CoveragePlot function was subsequently employed to visualize the MACS2 peaks in specific cell types against the 10x CellRanger peaks.

### Differential peaks motif analysis.

In Signac, we utilized the FindMarkers function to identify differentially expressed peaks among cells. Subsequently, the FindMotifs and MotifPlot functions were employed to compute and visualize differential motifs.

### GO analysis.

We performed GO enrichment analysis on treatment-associated DEGs using Metascape. The results were visualized with the ggplot2 R package (v.3.3.5).

### Pseudotime analysis.

Pseudotime analysis was performed using Monocle (v.2.22.0) to map the differentiation trajectory of CD4 T cell subtypes. The Seurat package’s VariableFeatures function identified highly variable genes as sorting genes.

### WGCNA.

WGCNA on scRNA-Seq data was conducted using the hdWGCNA package (v.0.2.26) following the official documentation (27).

### Cell-cell interaction analysis.

Communication analysis between immune cells and fibroblast subtypes was performed using the CellChat R package (v.1.6.1).

### Flow cytometry.

After treatment with lymphocyte separation solution (Solarbio, P8900 and P8620), PBMCs were collected from the interface between the PBS and Ficoll layers into new tubes. Live/dead cell counting was performed using AO/PI dye (Countstar, RE010212) with the Automated Fluorescence Cell Analyzer (Countstar Rigel S2), resulting in a live cell proportion of 93.3% to 98.2%. Notably, for intracellular cytokine staining, cells were stimulated for 5 hours with 50 ng/mL PMA (Sigma-Aldrich, 16561-29-8), 500 ng/mL ionomycin (Sigma-Aldrich, I3909), and GolgiPlug (BD Biosciences, 555029). Prior to incubation with intracellular or nuclear antibodies, cells were treated with a nuclear permeabilization kit (BioLegend, 424401) according to the manufacturer’s instructions, and Fc-block (BioLegend, 422302) was used for incubation. Subsequently, corresponding flow cytometry antibodies were incubated in flow cytometry staining buffer (Proteintech, PF00018) to detect specific cell protein in human or mouse cells. Antibodies used in Flow Cytometry experiments include: FOXP3 (BioLegend, 126419; BioLegend, 320014); CD4 (BioLegend, 100443; BioLegend, 300553); CD25 (BioLegend, 102051); IL-10 (BioLegend, 505007); TGF-β1 (BioLegend, 141403); TGF-β (BioLegend, 300004); CTLA4 (CD152) (BioLegend, 106305; BioLegend, 349905); CD44 (BioLegend, 103011); CD278 (ICOS) (BioLegend, 117419; BioLegend, 313507); KLRC1 (BioLegend, 142803; BioLegend, 375103); FGFBP2 (ATLAS, HPA039180); GZMB (CST, 65563S; CST, 50590S); T-bet (BioLegend, 644810); GNLY (BioLegend, 348003); IL-6 (BioLegend, 501106); TNF-α (BioLegend, 502909); IFN-γ (BioLegend, 502511); and IL-4 (BioLegend, 500808). Simultaneously, isotype control antibodies were used (BioLegend, 400525, 400411, 400418, 401903, 400607, 400111, 400907, 400611, 401207, 400113, 400139, 400101, 400407, 400141). Flow cytometry data were collected using the BD FACSCanto II flow cytometer (BD Biosciences), with single-color compensation for each fluorescence channel. Analysis was performed using FlowJo (v.10), where obvious dead cells and debris were excluded through forward scatter/side scatter (FSC/SSC) gating. Additionally, single cells were selected and aggregates and debris were excluded using FSC-height (FSC-H) and FSC-width (FSC-W) gating.

### Treg in in vitro suppression assays.

PBMCs from Healthy and GO donors were collected, and Treg were enriched using Treg beads (Miltenyi Biotec, 130-091-301). These cells were cultured for 7 days in a medium containing 5 mg/mL anti-CD3, 5 mg/mL anti-CD28, and 20 ng/mL recombinant IL-2 to promote expansion. CD8 T cells were isolated via flow cytometric sorting (BD FACSAria Fusion). To label CD8 T cells for in vitro suppression assays, 5 mM carboxyfluorescein succinimidyl ester (CFSE) (BioLegend, 423801) was prepared as a 5 μM working solution and incubated with the cells in the dark. CFSE-labeled CD8 T cells were cocultured with Treg at a 1:1 ratio in a medium containing 5 mg/mL anti-CD3 and 5 mg/mL anti-CD28 for 72 hours. Supernatants were collected for IFN-γ analysis using an ELISA kit (Elabscience, E-EL-H0108). After washing the cells with FACS buffer and centrifuging (450*g*), they were fixed in 1.6% PFA and analyzed for CFSE-labeled CD8 T cell proliferation using a BD FACSCanto II flow cytometer (BD Biosciences). Proliferation data were analyzed with FlowJo (v.10) within the lymphocyte population’s single-cell gate.

### qPCR.

High-quality RNA was extracted using the RNeasy Mini Kit (Qiagen, 74104) following the manufacturer’s instructions. Equal amounts of RNA were combined with specific primers and HiScript II One-Step qPCR SYBR Green Kit reaction solution (Vazyme, Q221) for qPCR, with expression values normalized to GAPDH levels. Primer sequences are provided in Supplemental Table 4.

### Construction of GO mouse model and corticosteroid treatment.

Following the method described by other researchers (34), we constructed a recombinant adenovirus AAV9-TSHR vector containing the human TSHR gene (1–289 aa). Female Balb/c mice were randomly divided into 2 groups and anesthetized with 0.5% pentobarbital. Each mouse was injected with a dose of 2 × 10^9^ viral particles into the tibialis anterior muscle. One group underwent GH model assessment after 3 injections, while the other group was assessed for the GO model after 6 injections. Assessment criteria included serum levels of T4, TSH, and thyroid receptor antibody (TRAb), as well as MRI. In the GH group, mice with elevated blood T4 levels, increased TSHR, decreased TSH levels, and negative MRI results (no substantial increase in orbital soft tissue volume) were considered successful GH models. In the GO group, mice with elevated blood T4 levels, increased TSHR, decreased TSH levels, and MRI evidence of increased orbital soft tissue volume were considered successful GO models. Successfully modeled mice were used for subsequent experiments. In the corticosteroid treatment experiment, the treatment group received i.p. injections of methylprednisolone at a dose of 1 mg/kg once a week, and mice were euthanized for tissue collection after 7 injections (35).

### ELISA.

Blood was collected from the orbital sinus of mice using a sterile needle and placed in 1.5 mL centrifuge tubes (Biosharp, BS-15-M) to stand for 1 hour. After centrifugation at 1,000*g* for 10 minutes, the supernatant was aspirated and stored at –80°C. Mouse serum TSH, T4, and TRAb levels were measured using ELISA kits from Shanghai Huding Biotechnology Co. Ltd.: TSH (catalog DB969-Mu), T4 (catalog DB725-Mu), and TRAb (catalog DB674-Mu), following the manufacturer’s instructions. The assays included sample dilution, addition to antigen-coated wells, washing, enzyme reagent addition, color development, termination, and optical density (OD) measurement at 450 nm.

### Ocular MRI.

Mouse orbital MRI T2 imaging was performed using the M3 series compact MRI system (Winsun Limited). The ImageJ software (NIH) was utilized with the Manager ROI function to measure the soft tissue volume in the mouse orbit. The software identified the edges of the orbital tissue, and after manual correction, calculated the area for each slice. The total volume was obtained by summing all slice areas and multiplying by the slice thickness used during MRI measurements.

### Vector construction.

*YY1* shRNA sequence (Supplemental Table 4) was cloned into lentivirus shRNA expression plasmid rLV-U6- YY1-shRNA-PGK-T2A-Puro-WPRE. cDNAs encoding *YY1* and *EGFP* were cloned into the rLV-CMV-YY1-T2A -WPRE/ rLV-CMV-EGFP-T2A-WPRE lentiviral plasmids. The plasmids were synthesized by BrainVTA Co. Ltd.

### Lentiviral lentivirus production.

Following the manufacturer’s instructions, 293FT cells (Invitrogen, R70007) were cultured in DMEM with 10% FBS and 400 μg/mL neomycin (BioShop, BL1303A). For transfection, 10 μg of the plasmid containing the vector, 10 μg of pMD2.G, and 15 μg of psPAX2 (Addgene) were mixed and transfected using 100 μL of Lipofectamine 2000 (Invitrogen, 1668027) and 200 μL of Plus Reagent (Invitrogen, 11514015). The media were changed 5 hours after transfection. Twenty-four hours later, the culture medium was collected and replaced with fresh medium. Virus supernatant was harvested 48 hours after transfection (6 μg/mL).

### Treg polarization in vitro and adoptive transfer experiment.

After euthanizing Balb/c mice, spleens were aseptically removed and homogenized with a 10 mL syringe, filtered, and subjected to red cell lysis. Following the manufacturer’s instructions, CD4 cells were isolated using CD4 beads (Miltenyi Biotec, 130-117-043). These cells were placed in a CD4 culture medium system containing 5 mg/mL anti-CD3 (BioLegend, 102115), 5 mg/mL anti-CD28 (BioLegend, 102115), and 20 ng/mL recombinant mouse IL-2 (Miltenyi Biotec, 130-120-662) and cultured for 3 days in a 5% CO_2_ incubator. After medium change, 5 ng/mL TGF-β (BioLegend, 781804) was added and cells were cultured for an additional 5 days. Subsequently, lentivirus carrying EGFP (MOI = 20) was added. After 8 hours, the medium was changed, and cells were cultured for another 3 days. Finally, Treg were isolated using Treg beads (Miltenyi Biotec, 130-091-041), resulting in purified EGFP-labeled Treg, which were then subjected to adoptive transfer experiments by tail vein injection of 1 × 10^6^ cells into GO or healthy mice.

### Treg collection and expansion in vitro.

Treg from healthy and patients with GO were enriched and expanded as previously described. Cells were then transfected with LV-YY1-shRNA and LV-YY1 (MOI = 20), with 5 μg/mL polybrene added. The medium was replaced 8 hours after transfection. After 2 days, cells received treatments with 25 μg/mL TSHR (Biotechne, 8950-TR), 50 ng/mL TNF-α (Peprotech, 00-01A), and 50 ng/mL IFN-γ (Peprotech, 300-02). RNA was extracted 2 days later.

### Primary cultures of OFs.

OFs were enriched and cultured based on a published protocol (54). After finely mincing tissue samples obtained from surgery, they were seeded onto a 24-well plate using DMEM containing 10% FBS (Thermo Fisher Scientific, 10099141C) and 1% penicillin/streptomycin (Thermo Fisher Scientific, 15140122). The 24-well plate was then incubated at 37°C with 5% CO_2_. After 3 days of incubation, OFs migrated out from the tissue fragments and reached confluence within 10 days. The adherent monolayer cells were passaged using 0.25% trypsin/EDTA to establish OFs cell lines.

### Cell coculture.

After preparing single-cell suspensions of OFs from healthy mice, PBMCs from healthy and GO mouse models were collected, and Treg were enriched using magnetic beads for 7 days. Cells were treated with 50 ng/mL TNF-α (Peprotech, 315-01A) and 25 μg/mL TSHR for 2 days before being centrifuged (450*g*). After discarding the culture medium, the cells were added to a DMEM culture system with mouse OFs and cocultured for 3 days. Cellular RNA was extracted for qPCR analysis, while cells in other plates were fixed with 4% PFA for immunofluorescence.

### Immunofluorescence.

Mouse periorbital connective tissue was extracted by overnight fixation in 4% paraformaldehyde (Biosharp, BL539A) at 4°C, followed by dehydration in 30% sucrose and embedding in Tissue-Tek O.C.T. Compound (Sakura Finetek). Tissues were frozen at –80°C, and 10 μm sections were cut using a Leica CM1860 cryostat (Leica Biosciences). In a 24-well plate for coculture, cells were fixed with 4% paraformaldehyde for 1 hour on coverslips, with all steps washed with PBS. Immunostaining antibodies included anti-GFP (Abcam, ab6673), anti-KLRC1 (Affinity, DF4808), anti–Collagen I (Abcam, ab34710), anti-α-SMA (Invitrogen, 53-9760-82), anti-Ki67 (Invitrogen, MA5-14520), and anti-APOE (Invitrogen, 701241), along with secondary antibodies (Invitrogen). Immunofluorescence images were captured using a ZEISS LSM 980 microscope, with analysis conducted in ImageJ software.

### Code availability.

The data analysis pipeline utilized in our study was outlined on the Seurat, Signac, and scRepertoire websites (https://satijalab.org/seurat; https://stuartlab.org/signac; https://www.borch.dev/uploads/screpertoire). No additional unique code was generated in this manuscript.

### Statistics.

We performed statistical analyses using GraphPad Prism (v.8.0) and R. All data types are nonnormally distributed. Therefore, all statistical analyses are conducted using nonparametric test. The box plots in the violin plots represent the median, 25th percentile and 75th percentile of the data. For comparisons involving more than 2 groups, *P* values were adjusted using the Bonferroni correction method. A P value less than or equal to 0.05 was considered statistically significant.

### Study approval.

This study obtained approval from the Ethics Committee of Zhongshan Ophthalmic Center (approval nos. 2019KYPJ114 and J2023201). The clinical trials number for this study is NCT03515863 (https://clinicaltrials.gov/). We followed the relevant ethical regulations for human research participants outlined in the Declaration of Helsinki. All participants were recruited from Zhongshan Ophthalmic Center, and written informed consent was obtained from each participant. All animal experimental procedures complied with relevant laws and regulations related to animal research.

### Data availability.

The scRNA-Seq, scATAC-Seq, and scTCR-Seq data of PBMC analyzed in this article are accessible under project accession no. PRJCA025456 as of the date of publication (https://ngdc.cncb.ac.cn/gsa/).

Data for the GO and healthy ocular tissue are sourced from the published database HRA000870, BD-related data are sourced from the database HRA004778, RA-related data are sourced from the database GSE208161, MS-related data are sourced from GSE138266, SLE-related data are sourced from GSE174188, and pSS-related data are sourced from GSE157278. All experimental data are available in the Supporting Data Values file.

## Author contributions

ZL and SRK designed the study, analyzed the single-cell data, and conducted the experiments. ZL took the lead in these efforts and is therefore designated as the first author. The manuscript was written by ZL and SRK and revised by ZXS. MZ prepared the figures and performed the statistical analyses. LS, YJH, and QHS assisted with the experiments. QKZ and CNP helped construct the mouse model. JSL, HSW, DLW, and RXC prepared the human PBMC samples. BX and XWL conducted MRI assessments in mice. ZM and XCL guided and coordinated the progress of the entire project and revised the manuscript. All authors read and approved the final manuscript.

## Supplementary Material

Supplemental data

Supplemental table 1

Supplemental table 2

Supplemental table 3

Supplemental table 4

Supplemental table 5

Supporting data values

## Version 1 10/04/2024

In-Press Preview

## Version 2 11/22/2024

Electronic publication
