# Supplementary material for: Dynamic transition of Tregs to cytotoxic phenotype amid systemic inflammation in Graves’ ophthalmopathy
Source: JCI Insight. 2024 Nov 22;9(22):e181488. doi: 10.1172/jci.insight.181488 (PMC11601897; doi:10.1172/jci.insight.181488)
Supplement: Supplemental data [file jciinsight-9-181488-s141.pdf]

Figure 6. Pathogenic role of Treg cytotoxic transition to the orbital lesions of GO
(A) Schematic diagram of the EGFP labeled autoreactive Treg adoptive transfer experiment.
(B) The proportion of EGFP+ KLRC1+ cells within CD4+ cells in the peripheral blood of Healthy and GO mouse after adoptive transfer experiment, representing the cytotoxic transition of Tregs in vivo (Healthy, n=3; GO, n=9). Data are represented as the median [IQR]. *P < 0.05 by Mann-Whitney U test.
(C) Immunofluorescence staining demonstrated the presence of KLRC1+ EGFP+ cells in the orbital tissues of GO model mice.
(D, E) The major subtypes of orbital tissues (D) and integrated CD4 T cells from PBMCs and tissues (E) based on scRNA-seq data of Healthy donors and GO patients; the upper-right UMAP plot illustrates the batch effects.
(F) The ligand-receptor interactions involving CD4 CTLs significantly altered in GO among three LPF subgroups, MYF, and COF. Red represents inflammation-regulating ligand-receptor interactions, while green indicates ligand-receptor interactions related to extracellular matrix remodeling.
(G) Experimental model of the pathogenic effect of Treg cytotoxic transformation on localised lesions in the orbit.
(H) Immunofluorescence showed the expression of specific proteins of OF cells in the co-culture system of healthy control and GO model mouse groups; The histogram on the right represents the quantitative statistics of these proteins. Data are represented as the median [IQR]. **P < 0.001 by Mann-Whitney U test.
(I) qRT-PCR histogram showed the expression of specific genes in cells of the co-culture system. Each group consists of three independent samples (each n = 6). Data are represented as the median [IQR]. **P < 0.001 by Mann-Whitney U test.
All experiments were repeated 3 times.

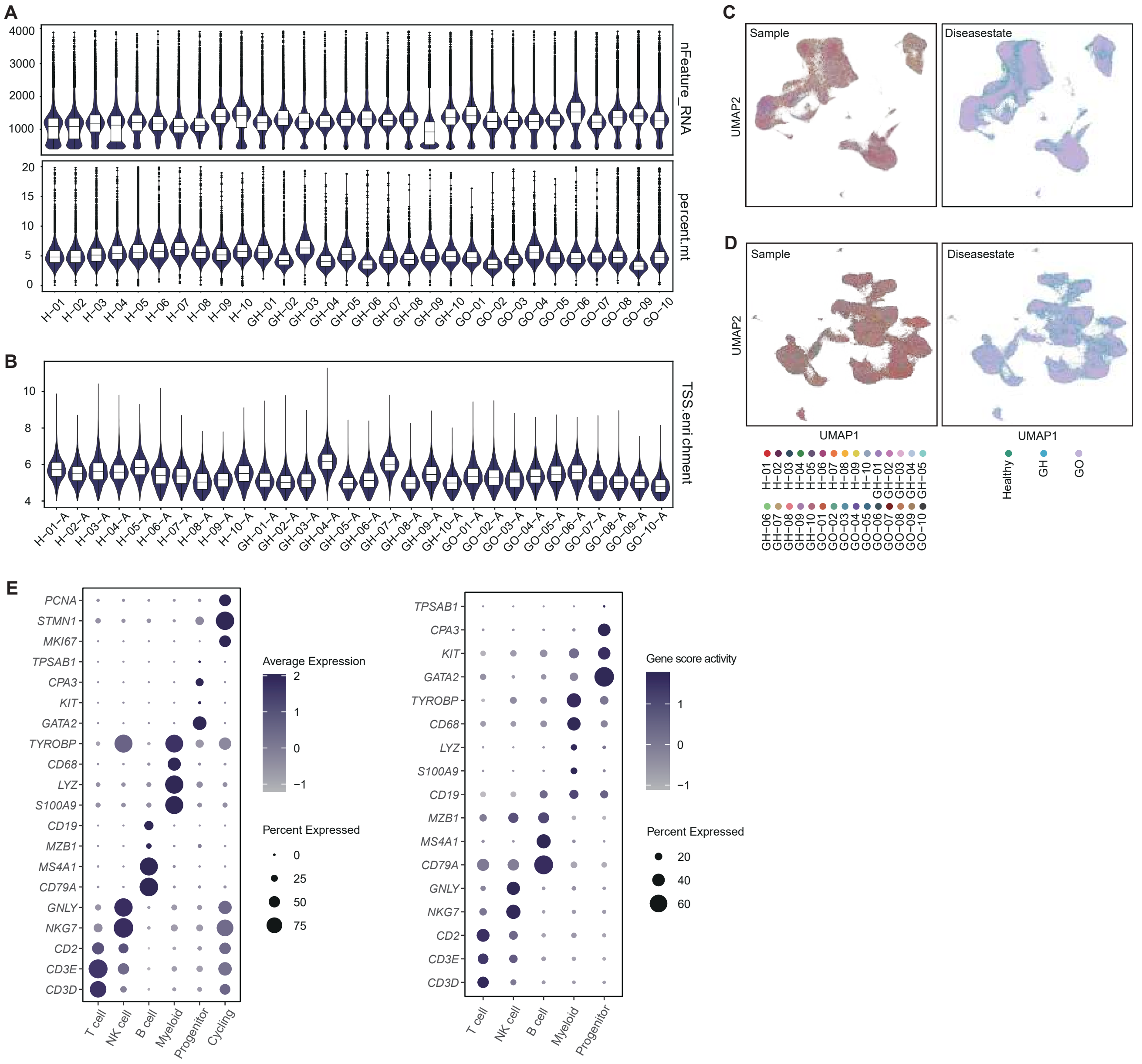
A
nFeature_RNA
percent.mt
H-01 H-02 H-03 H-04 H-05 H-06 H-07 H-08 H-09 H-10 GH-01 GH-02 GH-03 GH-04 GH-05 GH-06 GH-07 GH-08 GH-09 GH-10 GO-01 GO-02 GO-03 GO-04 GO-05 GO-06 GO-07 GO-08 GO-09 GO-10
B
TSS.enrichment
H-01-A H-02-A H-03-A H-04-A H-05-A H-06-A H-07-A H-08-A H-09-A H-10-A GH-01-A GH-02-A GH-03-A GH-04-A GH-05-A GH-06-A GH-07-A GH-08-A GH-09-A GH-10-A GO-01-A GO-02-A GO-03-A GO-04-A GO-05-A GO-06-A GO-07-A GO-08-A GO-09-A GO-10-A
C
Sample
Diseasestate
UMAP2
D
Sample
Diseasestate
UMAP2
UMAP1
UMAP1
Healthy
GH
GO
E
PCNA STMN1 MKI67 TPSAB1 CPA3 KIT GATA2 TYROBP CD68 LYZ S100A9 CD19 MZB1 MS4A1 CD79A GNLY NKG7 CD2 CD3E CD3D
T cell NK cell B cell Myeloid Progenitor Cycling
Average Expression
Percent Expressed
TPSAB1 CPA3 KIT GATA2 TYROBP CD68 LYZ S100A9 CD19 MZB1 MS4A1 CD79A GNLY NKG7 CD2 CD3E CD3D
T cell NK cell B cell Myeloid Progenitor
Gene score activity
Percent Expressed

Supplemental Figure 1. Quality control and batch effect removal in single-cell datasets
(A) Violin plots showed the distribution of the number of expressed genes and the percentage of mitochondrial RNA in each sample.
(B) Violin plots depicted TSS enrichment in all cells of each sample, with the addition of "-A" for each identical sample suffix for easy differentiation with scRNA-seq.
(C) UMAP representation of scRNA-seq after harmony correction on PCA dimensions, illustrating batch effects from sample batches, disease source batches.
(D) UMAP representation of scATAC-seq after harmony correction on LSI dimensions, showing batch effects from sample batches, disease source batches, and gender batches.
(E) Bubble plots of representative marker genes for all immune cell subtypes in the scRNA-seq and scATAC-seq datasets.

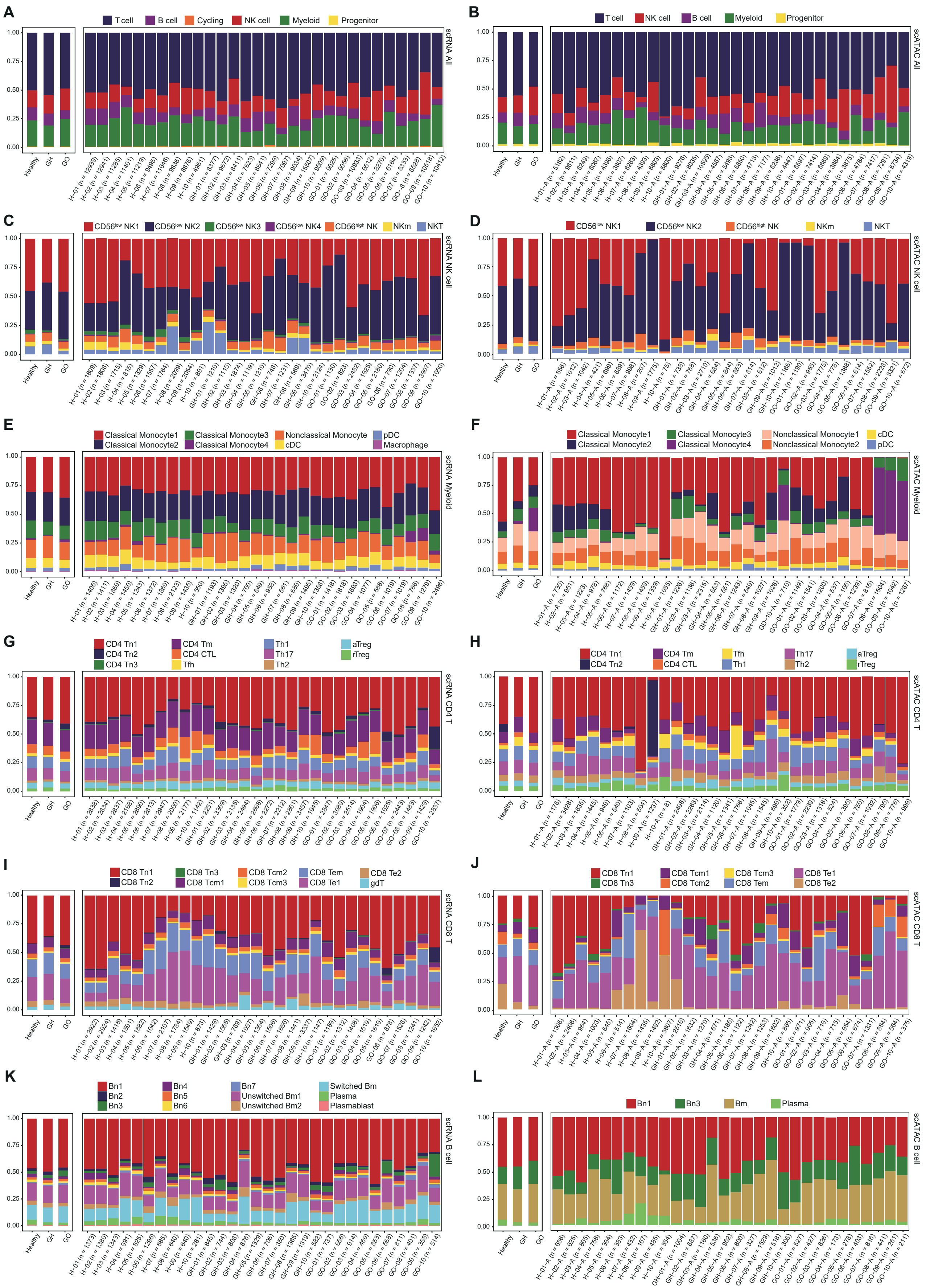

A
T cell
B cell
Cycling
NK cell
Myeloid
Progenitor
scRNA All
B
T cell
NK cell
B cell
Myeloid
Progenitor
scATAC All
C
CD56low NK1
CD56low NK2
CD56low NK3
CD56low NK4
CD56high NK
NKm
NKT
scRNA NK cell
D
CD56low NK1
CD56low NK2
CD56high NK
NKm
NKT
scATAC NK cell
E
Classical Monocyte1
Classical Monocyte2
Classical Monocyte3
Classical Monocyte4
Nonclassical Monocyte
cDC
pDC
Macrophage
scRNA Myeloid
F
Classical Monocyte1
Classical Monocyte2
Classical Monocyte3
Classical Monocyte4
Nonclassical Monocyte1
Nonclassical Monocyte2
cDC
pDC
scATAC Myeloid
G
CD4 Tn1
CD4 Tn2
CD4 Tn3
CD4 Tm
CD4 CTL
Tfh
Th1
Th17
Th2
aTreg
rTreg
scRNA CD4 T
H
CD4 Tn1
CD4 Tn2
CD4 Tm
CD4 CTL
Tfh
Th1
Th17
Th2
aTreg
rTreg
scATAC CD4 T
I
CD8 Tn1
CD8 Tn2
CD8 Tn3
CD8 Tcm1
CD8 Tcm2
CD8 Tcm3
CD8 Tem
CD8 Te1
CD8 Te2
gdT
scRNA CD8 T
J
CD8 Tn1
CD8 Tn3
CD8 Tcm1
CD8 Tcm2
CD8 Tcm3
CD8 Tem
CD8 Te1
CD8 Te2
scATAC CD8 T
K
Bn1
Bn2
Bn3
Bn4
Bn5
Bn6
Bn7
Unswitched Bm1
Unswitched Bm2
Switched Bm
Plasma
Plasmablast
scRNA B cell
L
Bn1
Bn3
Bm
Plasma
scATAC B cell
Healthy
GH
GO

Supplemental Figure 2. Breakdown of cellular composition in the study samples
(A, B) Stacked bar plots showed the proportion of each major immune cell subtype in scRNA-seq (A) and scATAC (B) datasets for each sample. Each column represents a group (left) or a sample (right), with different colors indicating distinct cell types within the samples.
(C, D) Stacked bar plots illustrated the proportion of each NK cell subtype in scRNA-seq (C) and scATAC (D) datasets for each sample. Each column represents a group (left) or a sample (right), with different colors representing various cell types within the samples.
(E, F) Stacked bar plots displayed the proportion of each myeloid cell subtype in scRNA-seq (E) and scATAC (F) datasets for each sample. Each column represents a group (left) or a sample (right), with different colors indicating diverse cell types within the samples.
(G, H) Stacked bar plots indicated the proportion of each CD4 T cell subtype in scRNA-seq (G) and scATAC (H) datasets for each sample. Each column represents a group (left) or a sample (right), with different colors representing distinct cell types within the samples.
(I, J) Stacked bar plots represented the proportion of each CD8 T cell subtype in scRNA-seq (I) and scATAC (J) datasets for each sample. Each column represents a group (left) or a sample (right), with different colors indicating various cell types within the samples.
(K, L) Stacked bar plots depicted the proportion of each B cell subtype in scRNA-seq (K) and scATAC (L) datasets for each sample. Each column represents a group (left) or a sample (right), with different colors representing diverse cell types within the samples.

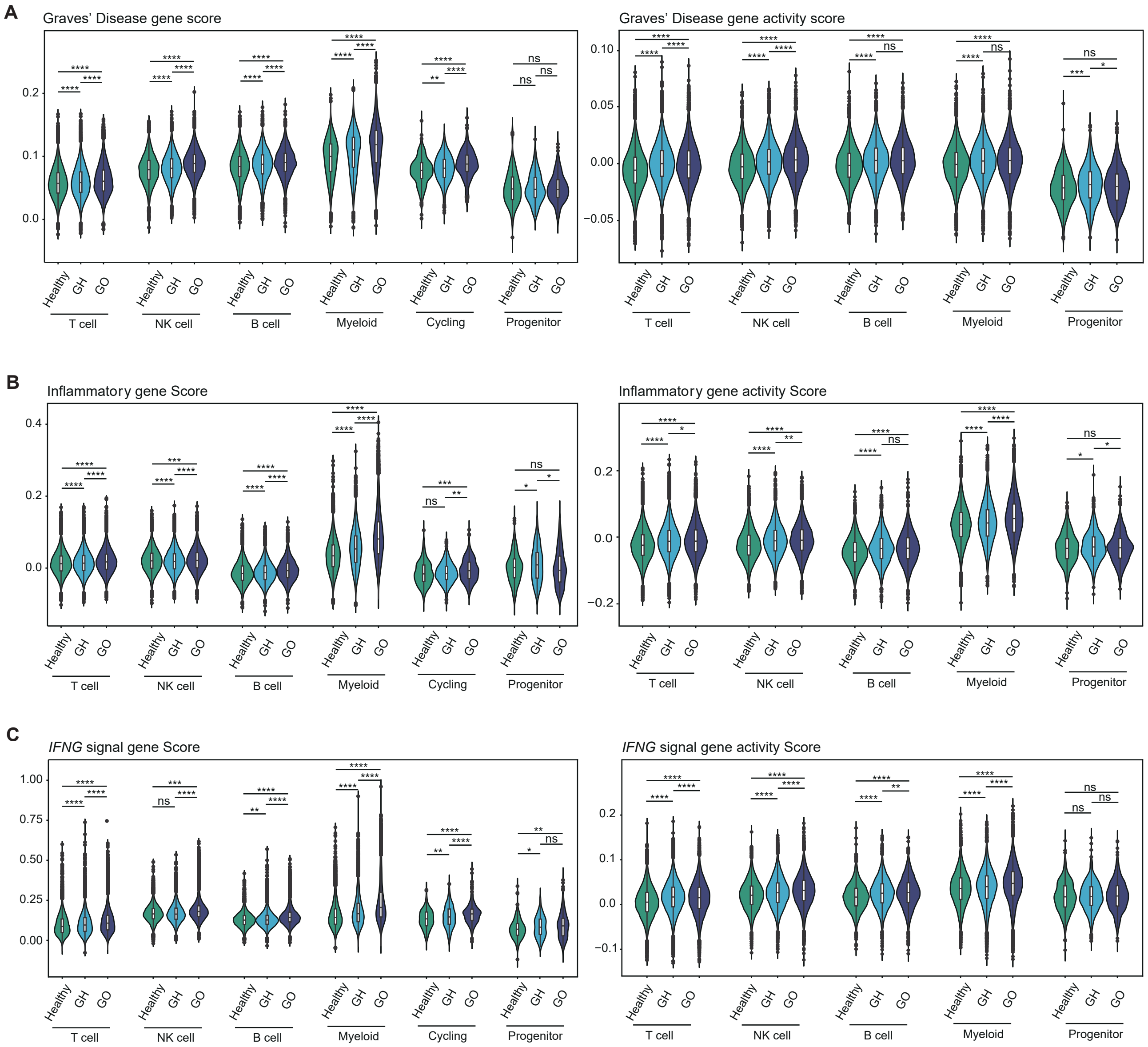
A
Graves' Disease gene score
Graves' Disease gene activity score
B
Inflammatory gene Score
Inflammatory gene activity Score
C
IFNG signal gene Score
IFNG signal gene activity Score
Healthy
GH
GO
T cell
NK cell
B cell
Myeloid
Cycling
Progenitor

Supplemental Figure 3. Features of inflammatory immune environment within GO progression
(A) Violin plots displayed the gene expression and activity scores of the Graves' Disease gene set in major immune cell subtypes in the Healthy, GH, and GO groups. Data are represented as the median [IQR]. *P.adj < 0.05, ****P.adj < 0.00001 by Mann-Whitney U test.
(B) Violin plots illustrated the gene expression and activity scores of the Inflammatory gene set in major immune cell subtypes in the Healthy, GH, and GO groups. Data are represented as the median [IQR]. *P.adj < 0.05, ****P.adj < 0.00001 by Mann-Whitney U test.
(C) Violin plots presented the gene expression and activity scores of the IFNG signal gene set in major immune cell subtypes in the Healthy, GH, and GO groups. Data are represented as the median [IQR]. *P.adj < 0.05, ****P.adj < 0.00001 by Mann-Whitney U test.

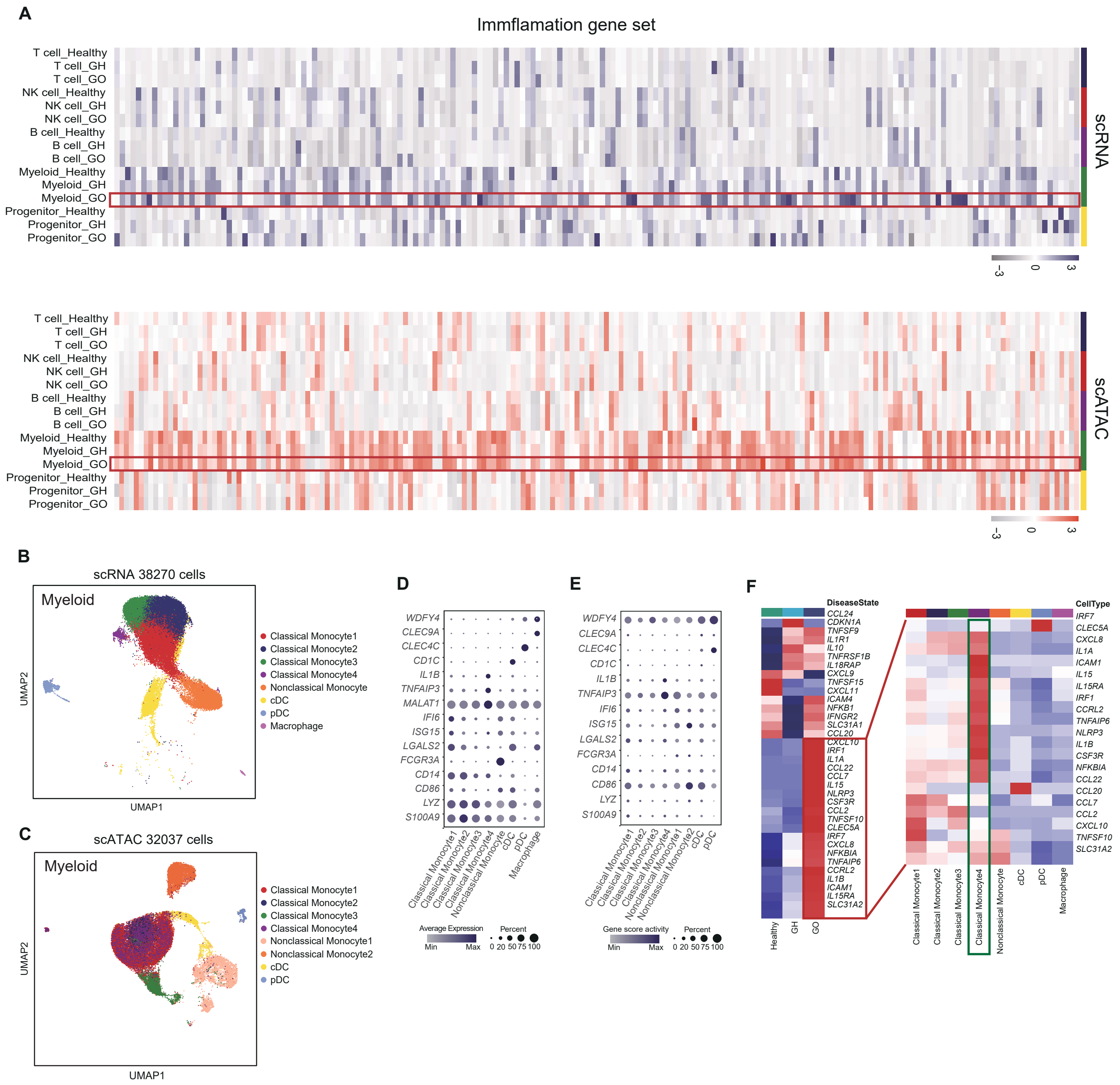
A
Immflamation gene set
T cell_Healthy
T cell_GH
T cell_GO
NK cell_Healthy
NK cell_GH
NK cell_GO
B cell_Healthy
B cell_GH
B cell_GO
Myeloid_Healthy
Myeloid_GH
Myeloid_GO
Progenitor_Healthy
Progenitor_GH
Progenitor_GO
scRNA
scATAC
-3 0 3
B
scRNA 38270 cells
Myeloid
UMAP2
UMAP1
Classical Monocyte1
Classical Monocyte2
Classical Monocyte3
Classical Monocyte4
Nonclassical Monocyte
cDC
pDC
Macrophage
C
scATAC 32037 cells
Myeloid
Classical Monocyte1
Classical Monocyte2
Classical Monocyte3
Classical Monocyte4
Nonclassical Monocyte1
Nonclassical Monocyte2
cDC
pDC
D
WDFY4
CLEC9A
CLEC4C
CD1C
IL1B
TNFAIP3
MALAT1
IFI6
ISG15
LGALS2
FCGR3A
CD14
CD86
LYZ
S100A9
Average Expression
Min Max
Percent
0 20 50 75 100
E
Gene score activity
F
DiseaseState
Healthy
GH
GO
CCL24
CDKN1A
TNFSF9
IL1R1
IL10
TNFRSF1B
IL18RAP
ICAM4
CXCL9
TNFSF15
CXCL11
NFKB1
IFNGR2
SLC31A1
CCL20
CXCL10
IRF1
IL1A
CCL22
CCL7
IL15
NLRP3
CSF3R
CCL2
TNFSF10
CLEC5A
IRF7
CXCL8
NFKBIA
TNFAIP6
CCRL2
IL1B
ICAM1
IL15RA
SLC31A2
CellType
IRF7
CLEC5A
CXCL8
IL1A
ICAM1
IL15
IL15RA
IRF1
CCRL2
TNFAIP6
NLRP3
IL1B
CSF3R
NFKBIA
CCL22
CCL20
CCL7
CCL2
CXCL10
TNFSF10
SLC31A2
Classical Monocyte1
Classical Monocyte2
Classical Monocyte3
Classical Monocyte4
Nonclassical Monocyte
cDC
pDC
Macrophage

Supplemental Figure 4. Characterization of Myeloid subsets during GO progression
(A) Heatmaps illustrated the expression (up) and gene activity score (down) of Immflamation response gene (GSEA: M5932) among major cell types.
(B, C) UMAP plots depicted the clustering of all myeloid cell subtype in scRNA-seq (B) and scATAC-seq (C) datasets.
(D, E) Representative bubble plots of marker genes for each myeloid cell subtype in scRNA-seq (D) and scATAC-seq (E) datasets.
(F) Heatmap showed the expression levels of typical inflammatory genes in myeloid cells from Healthy, GH, and GO groups, as well as in each myeloid cell subtype.

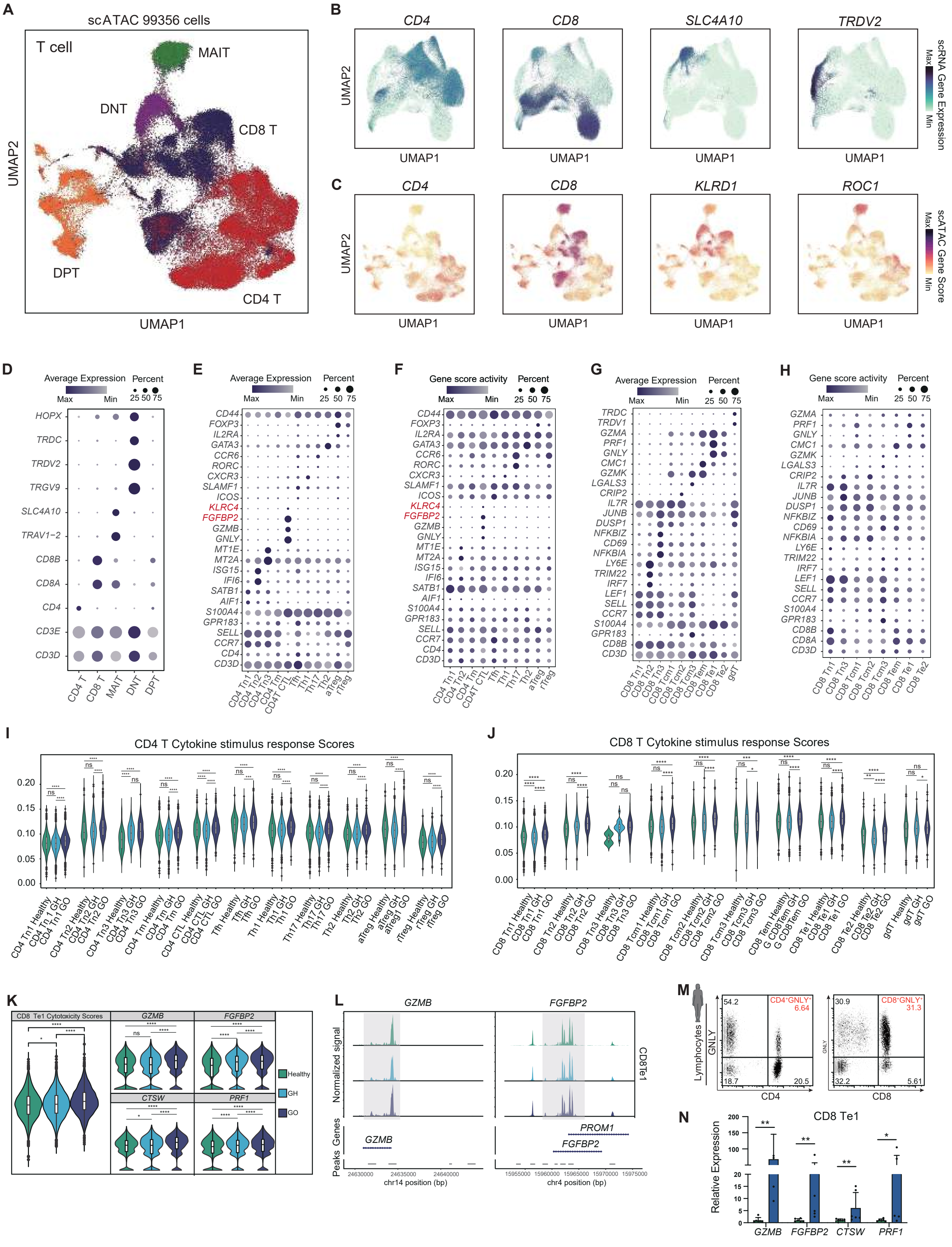
A
scATAC 99356 cells
T cell
MAIT
DNT
CD8 T
DPT
CD4 T
UMAP1
UMAP2
B
CD4
CD8
SLC4A10
TRDV2
scRNA Gene Expression
Max
Min
C
CD4
CD8
KLRD1
ROC1
scATAC Gene Score
D
Average Expression
Percent
HOPX
TRDC
TRDV2
TRGV9
SLC4A10
TRAV1−2
CD8B
CD8A
CD4
CD3E
CD3D
CD4 T
CD8 T
MAIT
DNT
DPT
E
F
Gene score activity
KLRC4
FGFBP2
G
H
I
CD4 T Cytokine stimulus response Scores
J
CD8 T Cytokine stimulus response Scores
K
CD8 Te1 Cytotoxicity Scores
GZMB
FGFBP2
CTSW
PRF1
Healthy
GH
GO
L
Normalized signal
Peaks Genes
CD8Te1
PROM1
chr14 position (bp)
chr4 position (bp)
M
Lymphocytes
GNLY
CD4⁺GNLY⁺
CD8⁺GNLY⁺
N
CD8 Te1
Relative Expression

Supplemental Figure 5. Annotation of T-cell subsets in single-cell datasets
(A) UMAP plot displayed the clustering of major T-cell subtypes in the scATAC-seq dataset.
(B, C) UMAP plots illustrated the expression levels (B) and gene activity scores (C) of typical marker genes for major T-cell subtypes.
(D) Bubble plot of representative marker genes for major T-cell subtypes in the scRNA-seq dataset.
(E, F) Bubble plots of representative marker genes for all CD4 T-cell subtypes in the scRNA-seq (E) and scATAC-seq (F) datasets.
(G, H) Bubble plots of representative marker genes for all CD8 T-cell subtypes in the scRNA-seq (G) and scATAC-seq (H) datasets.
(I, J) The expression scores of the CD4 T Cytokine stimulus response gene set in CD4 T-cell subtypes (I) and the CD8 T Cytokine stimulus response gene set in CD8 T-cell subtypes (J) from Healthy, GH, and GO groups. Data are represented as the median [IQR]. *P.adj < 0.05, ****P.adj < 0.00001 by Mann-Whitney U test.
(K) The expression scores for the Cytotoxicity functional gene set and the gene expression levels of GZMB, FGFBP2, CTSW, and PRF1 in CD8 Te1 from Healthy, GH, and GO groups. Data are represented as the median [IQR]. *P.adj < 0.05, ****P.adj < 0.00001 by Mann-Whitney U test.
(L) Chromatin accessibility peak plots for GZMB and FGFBP2 genes in CD8 Te1 from Healthy, GH, and GO groups.
(M) Flow cytometry plots illustrated the sorting strategy for human PBMCs into CD4 CTL and GNLY+ CD8 T cells.
(N) Bar graphs from qRT-PCR showed the gene expression levels of GZMB, FGFBP2, CTSW, and PRF1 in CD8 Te1 from peripheral blood of Healthy and GO donors (Healthy, n=6; GO, n=6). Data are represented as the median [IQR]. *P < 0.05, **P < 0.001 by Mann-Whitney U test. The experiments were repeated 3 times.

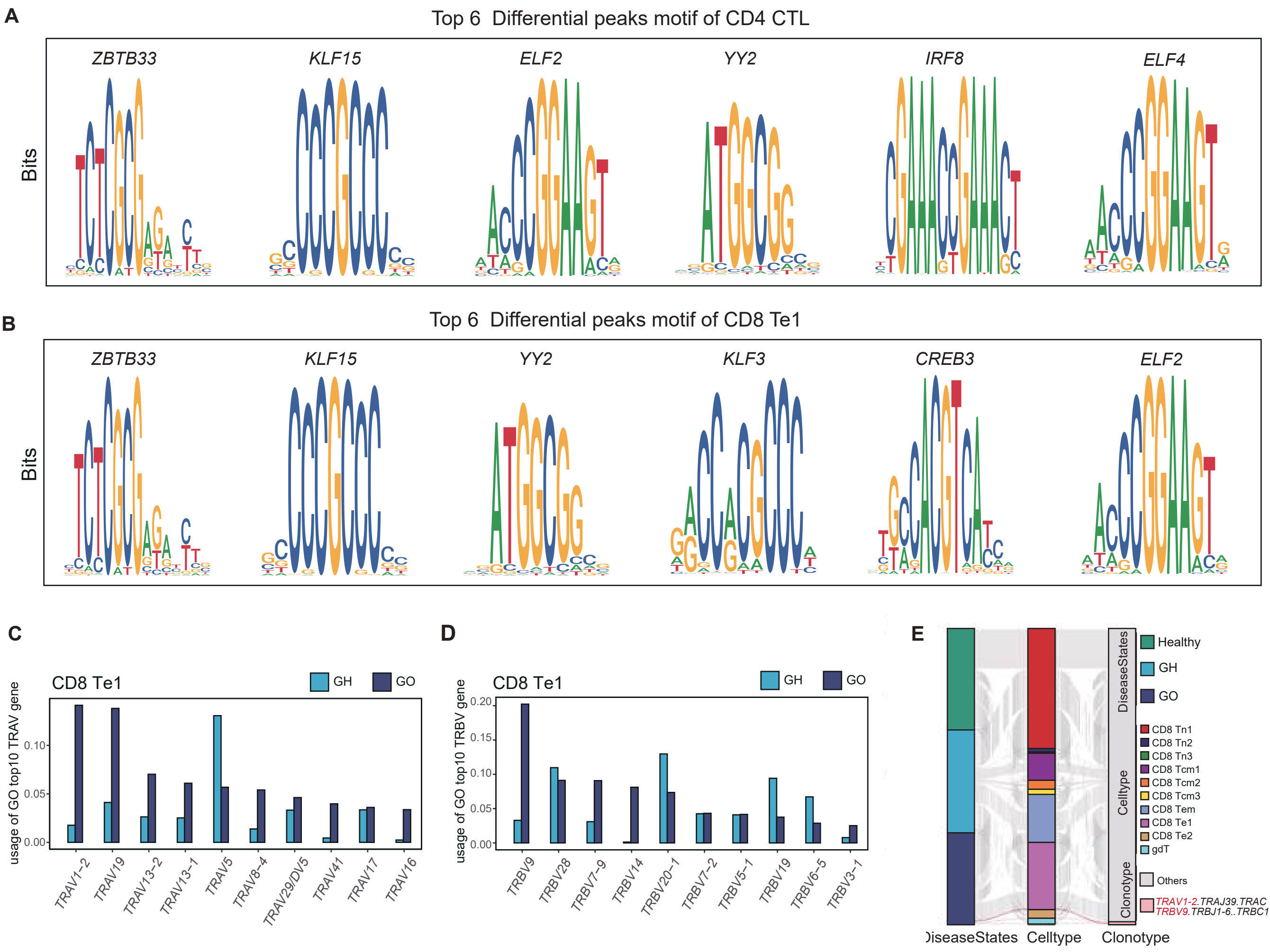
A
Top 6 Differential peaks motif of CD4 CTL
ZBTB33
KLF15
ELF2
YY2
IRF8
ELF4
Bits
B
Top 6 Differential peaks motif of CD8 Te1
ZBTB33
KLF15
YY2
KLF3
CREB3
ELF2
Bits
C
CD8 Te1
GH
GO
usage of GO top10 TRAV gene
0.10
0.05
0.00
TRAV1-2
TRAV19
TRAV13-2
TRAV13-1
TRAV5
TRAV8-4
TRAV29/DV5
TRAV41
TRAV17
TRAV16
D
CD8 Te1
GH
GO
usage of GO top10 TRBV gene
0.20
0.15
0.10
0.05
0.00
TRBV9
TRBV28
TRBV7-9
TRBV14
TRBV20-1
TRBV7-2
TRBV5-1
TRBV19
TRBV6-5
TRBV3-1
E
DiseaseStates
Celltype
Clonotype
Healthy
GH
GO
CD8 Tn1
CD8 Tn2
CD8 Tn3
CD8 Tcm1
CD8 Tcm2
CD8 Tcm3
CD8 Tem
CD8 Te1
CD8 Te2
gdT
Others
TRAV1-2.TRAJ39.TRAC
TRBV9.TRBJ1-6..TRBC1

Supplemental Figure 6. Analysis of GO-specific TF and antigen clone epitopes
(A, B) Top 6 differential peaks motif analysis of in CD4 CTL (A) and CD8 Te1 (B) cells between GH and GO groups.
(C, D) Bar charts illustrated the expression levels of the top 10 GO-specific TRAV genes (G) and top 10 TRBV genes (H) in CD8 T-cell subtypes from Healthy, GH, and GO groups.
(E) Sankey diagram represented the relationship between clonotype genes in CD8 T-cell subtypes from Healthy, GH, and GO groups. GO-specific TCR are highlighted

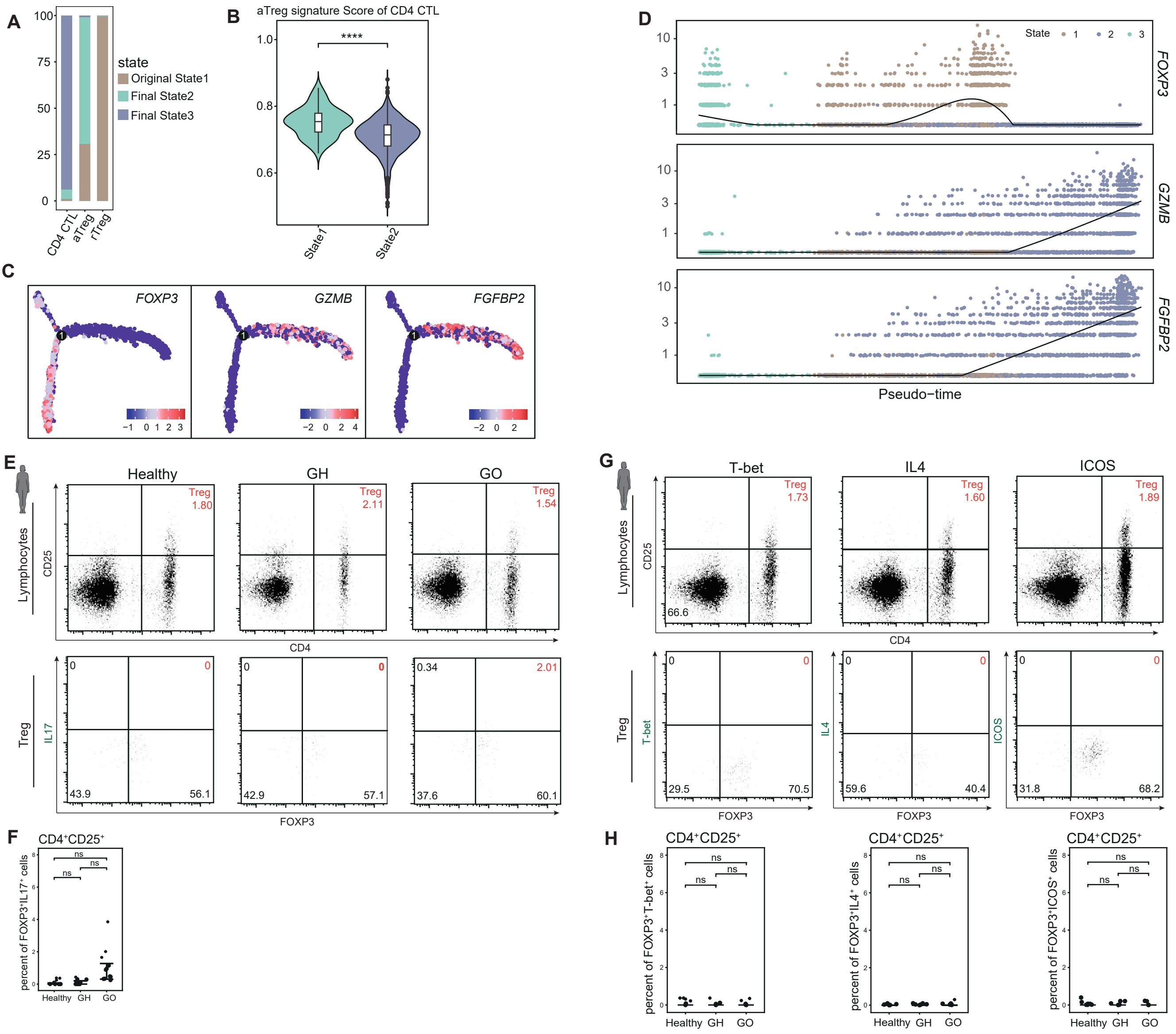
A
B
aTreg signature Score of CD4 CTL
state
Original State1
Final State2
Final State3
CD4 CTL
aTreg
rTreg
State1
State2
C
FOXP3
GZMB
FGFBP2
D
State
FOXP3
GZMB
FGFBP2
Pseudo−time
E
Healthy
GH
GO
Lymphocytes
CD25
CD4
Treg
IL17
FOXP3
G
T-bet
IL4
ICOS
F
CD4⁺CD25⁺
percent of FOXP3⁺IL17⁺ cells
H
percent of FOXP3⁺T-bet⁺ cells
percent of FOXP3⁺IL4⁺ cells
percent of FOXP3⁺ICOS⁺ cells

Supplemental Figure 7. Detailed analysis of Treg during GO progression
(A) Stacked bar charts illustrated the proportions of CD4 CTL, aTreg, and rTreg at different time points.
(B) Violin plots depicted the expression scores of transcriptionally characteristic genes for aTreg in CD4 CTL from State1 and State2. Data are represented as the median [IQR]. *P.adj < 0.05, ****P.adj < 0.00001 by Mann-Whitney U test.
(C, D) Expression profiles of FOXP3, GZMB, and FGFBP2 genes across cells along a pseudotime trajectory.
(E, F) Flow cytometry plots and dot plots illustrated the expression of IL17 in CD4+ CD25+ FOXP3+ Treg from peripheral blood in Healthy, GH, and GO groups (Healthy, n=9; GH, n=9; GO, n=9). Data are represented as the median [IQR].
*P.adj < 0.05 < 0.05 by Mann-Whitney U test.
(G, H) Flow cytometry plots and dot plots displayed the expression of T-bet, IL4, and ICOS in CD4+ CD25+ FOXP3+ Treg from the GO group in peripheral blood (Healthy, n=9; GH, n=9; GO, n=9). Data are represented as the median [IQR].
*P.adj < 0.05 by Mann-Whitney U test.

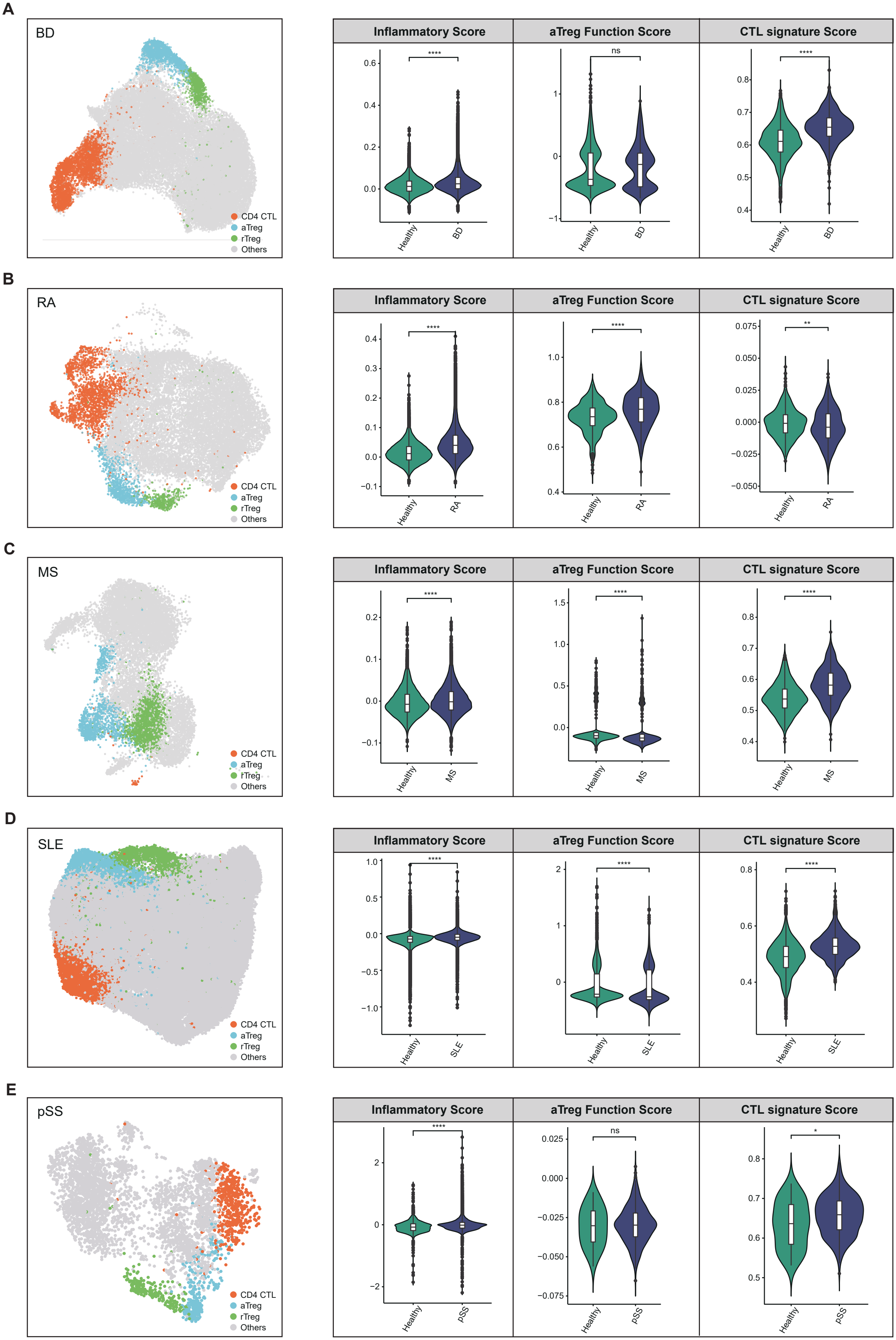
A
BD
CD4 CTL
aTreg
rTreg
Others
Inflammatory Score
aTreg Function Score
CTL signature Score
Healthy
BD
****
ns
B
RA
CD4 CTL
aTreg
rTreg
Others
Inflammatory Score
aTreg Function Score
CTL signature Score
Healthy
RA
****
**
C
MS
CD4 CTL
aTreg
rTreg
Others
Inflammatory Score
aTreg Function Score
CTL signature Score
Healthy
MS
****
D
SLE
CD4 CTL
aTreg
rTreg
Others
Inflammatory Score
aTreg Function Score
CTL signature Score
Healthy
SLE
****
E
pSS
CD4 CTL
aTreg
rTreg
Others
Inflammatory Score
aTreg Function Score
CTL signature Score
Healthy
pSS
****
ns
*

Supplemental Figure 8. Observation of abnormal aTreg with elevated CTL transcriptional signature in other autoimmune diseases
(A) Left: UMAP of CD4 CTL, aTreg, and rTreg in BD. Right: Violin plots depicting the overall inflammatory levels and the expression scores of the aTreg functional gene set and CD4 CTL transcriptional feature gene set in both healthy and BD groups. Data are represented as the median [IQR]. *P.adj < 0.05, ****P.adj < 0.00001 by Mann-Whitney U test.
(B) Left: UMAP of CD4 CTL, aTreg, and rTreg in RA. Right: Violin plots displaying the overall inflammatory levels and the expression scores of the aTreg functional gene set and CD4 CTL transcriptional feature gene set in both healthy and RA groups. Data are represented as the median [IQR]. *P.adj < 0.05, ****P.adj < 0.00001 by Mann-Whitney U test.
(C) Left: UMAP of CD4 CTL, aTreg, and rTreg in MS. Right: Violin plots presenting the overall inflammatory levels and the expression scores of the aTreg functional gene set and CD4 CTL transcriptional feature gene set in both healthy and MS groups. Data are represented as the median [IQR]. *P.adj < 0.05, ****P.adj < 0.00001 by Mann-Whitney U test.
(D) Left: UMAP of CD4 CTL, aTreg, and rTreg in SLE. Right: Violin plots showing the overall inflammatory levels and the expression scores of the aTreg functional gene set and CD4 CTL transcriptional feature gene set in both healthy and SLE groups.
(E) Left: UMAP of CD4 CTL, aTreg, and rTreg in pSS. Right: Violin plots indicating the overall inflammatory levels and the expression scores of the aTreg functional gene set and CD4 CTL transcriptional feature gene set in both healthy and pSS groups. Data are represented as the median [IQR]. *P.adj < 0.05, ****P.adj < 0.00001 by Mann-Whitney U test.

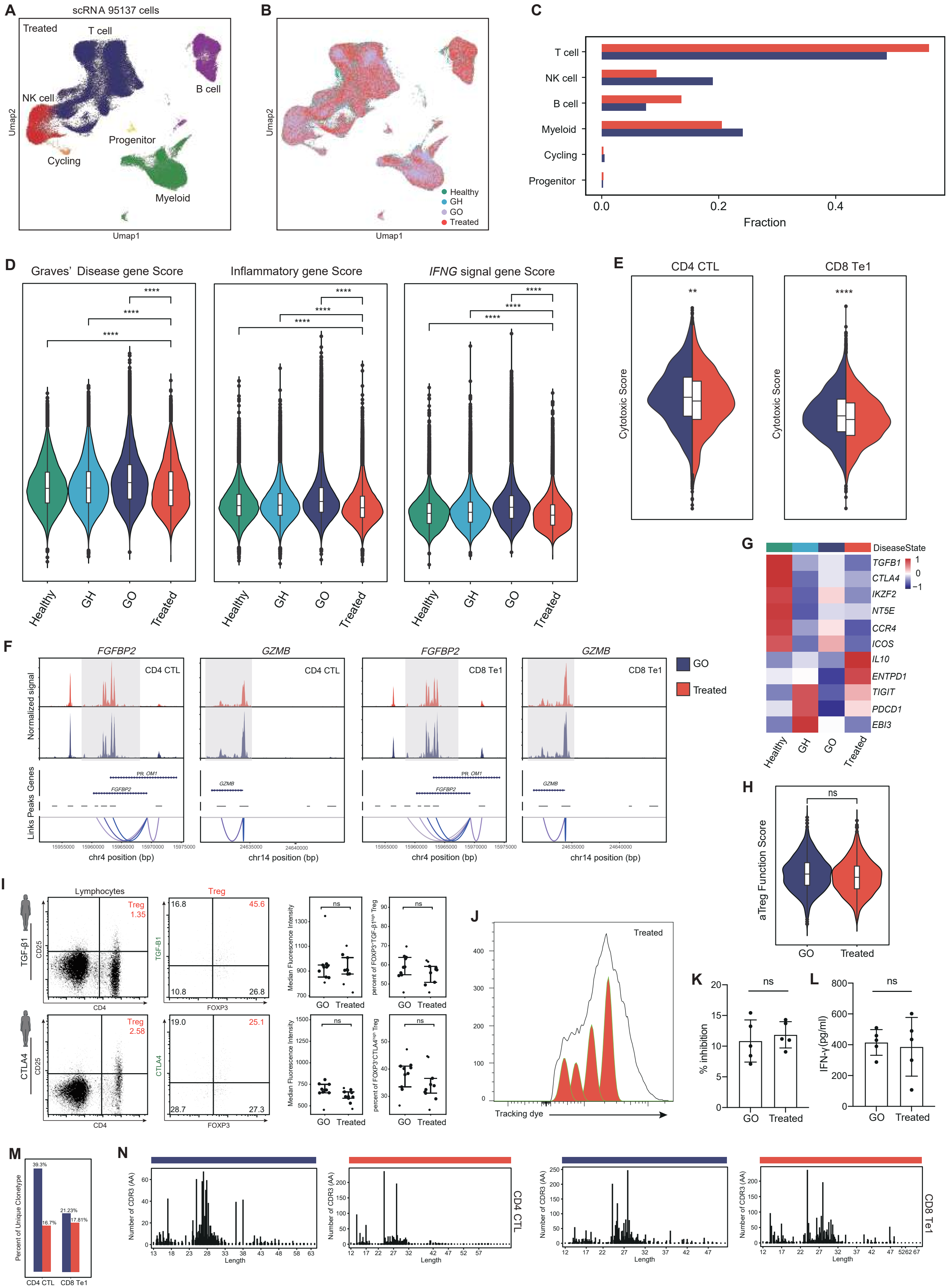

A
scRNA 95137 cells
Treated
T cell
B cell
NK cell
Progenitor
Cycling
Myeloid
Umap1
Umap2
B
Healthy
GH
GO
Treated
C
T cell
NK cell
B cell
Myeloid
Cycling
Progenitor
Fraction
D
Graves' Disease gene Score
Inflammatory gene Score
IFNG signal gene Score
Healthy
GH
GO
Treated
E
CD4 CTL
CD8 Te1
Cytotoxic Score
F
FGFBP2
GZMB
CD4 CTL
CD8 Te1
GO
Treated
Normalized signal
Links Peaks Genes
chr4 position (bp)
chr14 position (bp)
G
DiseaseState
TGFB1
CTLA4
IKZF2
NT5E
CCR4
ICOS
IL10
ENTPD1
TIGIT
PDCD1
EBI3
H
aTreg Function Score
ns
I
Lymphocytes
Treg
TGF-β1
CTLA4
CD4
CD25
FOXP3
Median Fluorescence Intensity
J
Treated
Tracking dye
K
% inhibition
L
IFN-γ(pg/ml)
M
Percent of Unique Clonetype
CD4 CTL
CD8 Te1
N
Number of CDR3 (AA)
Length

Supplemental Figure 9. Effects of corticosteroid therapy on patients with GO
(A) The distribution of major immune cell subtypes of the scRNA-seq dataset in Treated group.
(B) UMAP represented batch effects from four groups.
(C) The proportions of major immune cell subtypes.
(D) The expression scores of Graves' Disease, Inflammatory, and IFNG signal genes. Data are represented as the median [IQR]. ****P.adj < 0.00001 by Mann-Whitney U test.
(E) The expression scores of cytotoxic genes in CD4 CTL and CD8 Te1 cells in the GO and Treated groups. Data are represented as the median [IQR]. ****P < 0.00001 by Mann-Whitney U test.
(F) Chromatin accessibility plots for FGFBP2 and GZMB genes in CD4 CTL and CD8 Te1 cells between the two groups.
(G) The expression levels of key treg's functional genes in aTreg.
(H) The aTreg function score of GO and Treated groups. Data are represented as the median [IQR]. *P < 0.05 by Mann-Whitney U test.
(I) The expression levels and proportions of TGF-β1 and CTLA4 from human GO and Treated groups (GO, n=9; Treated, n=9). Data are represented as the median [IQR]. *P < 0.05 by Mann-Whitney U test. The experiments were repeated 3 times.
(J) The proliferation of CD8+ T cells co-cultured with Treg cells of Treated group.
(K) The inhibition of CD8+ T cells co-cultured with Treg cells (GO, n=5; Treated, n=5). Data are represented as the median [IQR]. *P < 0.05 by Mann-Whitney U test.
(L) The IFN-γ production of CD8+ T cells co-cultured with Treg cells (GO, n=5; Treated, n=5). Data are represented as the median [IQR]. *P < 0.05 by Mann-Whitney U test.The experiments were repeated 3 times.
(M) The unique clonal proportions of CD4 CTL and CD8 Te1 cells in the two groups.
(N) Composition of CD3 amino acids in CD4 CTL and CD8 Te1 in the two groups.

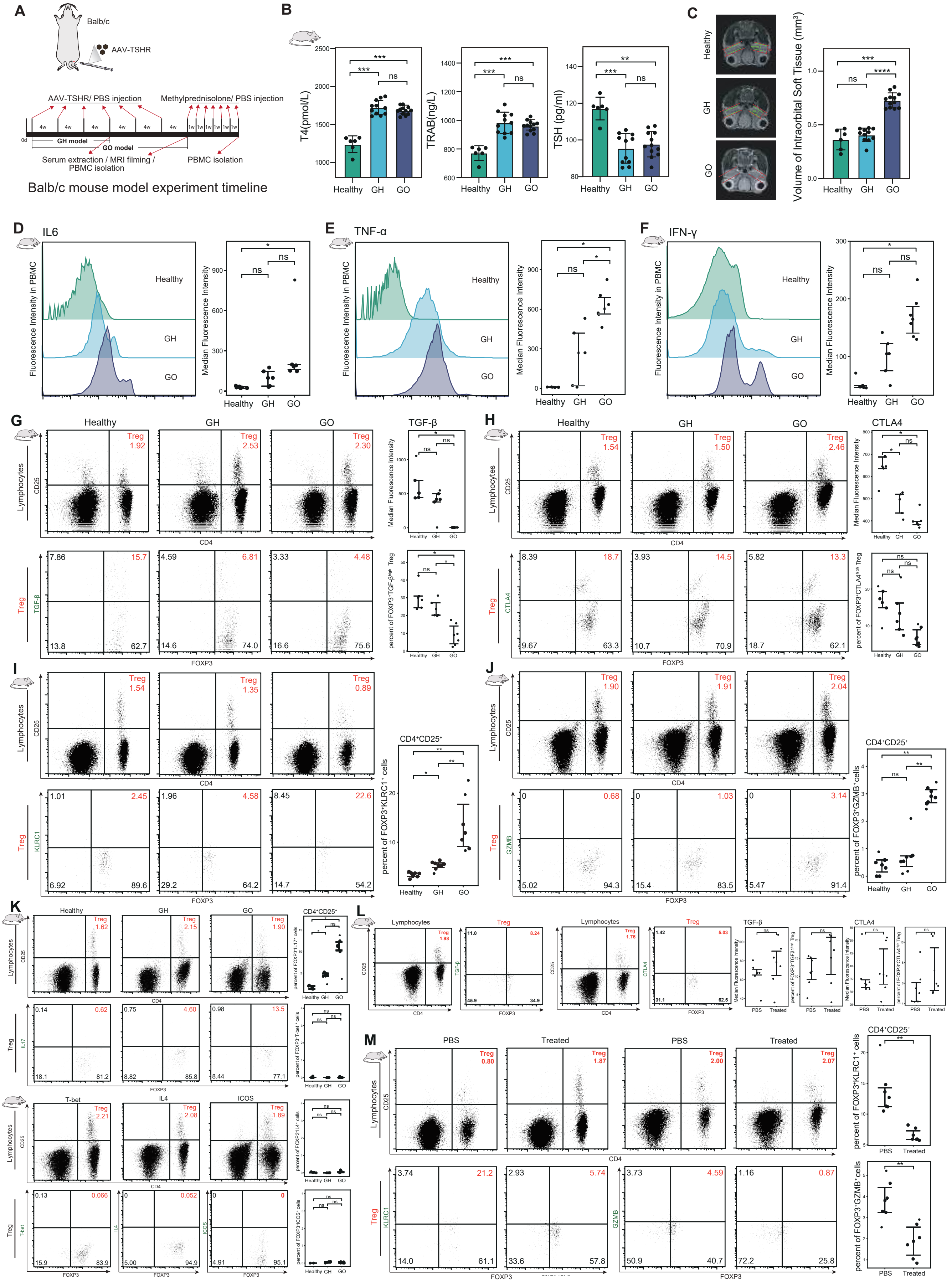

A
Balb/c
AAV-TSHR
AAV-TSHR/ PBS injection
Methylprednisolone/ PBS injection
GH model
GO model
Serum extraction / MRI filming / PBMC isolation
PBMC isolation
Balb/c mouse model experiment timeline
B
T4(pmol/L)
TRAB(ng/L)
TSH (pg/ml)
Healthy
GH
GO
C
Volume of Intraorbital Soft Tissue (mm³)
D
IL6
E
TNF-α
F
IFN-γ
Fluorescence Intensity in PBMC
Median Fluorescence Intensity
G
TGF-β
H
CTLA4
Lymphocytes
Treg
CD25
CD4
FOXP3
I
J
CD4⁺CD25⁺
percent of FOXP3⁺KLRC1⁺ cells
percent of FOXP3⁺GZMB⁺ cells
KLRC1
GZMB
K
L
M
PBS
Treated

Supplemental Figure 10. Similarities of systemic inflammation and Treg characteristics between the GO mouse model and human GO patients

(A) The pipeline of the GO mouse model and the specific experimental time points.

(B, C) Criteria for determining the model in mouse: the expression of T4, TRAB and TSH (B), and the volume of orbital soft tissue (C) (Healthy, n=6; GH, n=11; GO, n=12). Data are represented as the median [IQR]. ****P.adj < 0.00001 by Mann-Whitney U test.

(D, E, F) The expression levels of IL6 (D), TNF-α (E), and IFN-γ (F) of Healthy, GH, and GO mouse (Healthy, n=5; GH, n=5; GO, n=6). Data are represented as the median [IQR]. *P.adj < 0.05 by Mann-Whitney U test.

(G, H) The expression levels of TGF-β (G) and CTLA4 (H) from three groups (Healthy, n=5; GH, n=5; GO, n=6). Data are represented as the median [IQR]. *P.adj < 0.05 by Mann-Whitney U test.

(I, J) The expression levels of KLRC1 (I) and GZMB (J) from three groups (Healthy, n=7; GH, n=7; GO, n=7). Data are represented as the median [IQR]. *P.adj < 0.05, **P < 0.001 by Mann-Whitney U test.

(K) The expression levels of IL17 (top) and the expression levels of T-bet, IL4, and ICOS (bottom) from three groups (Healthy, n=5; GH, n=5; GO, n=6). Data are represented as the median [IQR]. *P.adj < 0.05 by Mann-Whitney U test.

(L) The expression levels of TGF-β and CTLA4 from GO and Treated groups (GO, n=6; Treated, n=6). Data are represented as the median [IQR]. *P.adj < 0.05 by Mann-Whitney U test.

(M) The expression levels of KLRC1 and GZMB from GO and Treated groups (GO, n=6; Treated, n=6). Data are represented as the median [IQR]. **P.adj < 0.001 by Mann-Whitney U test.

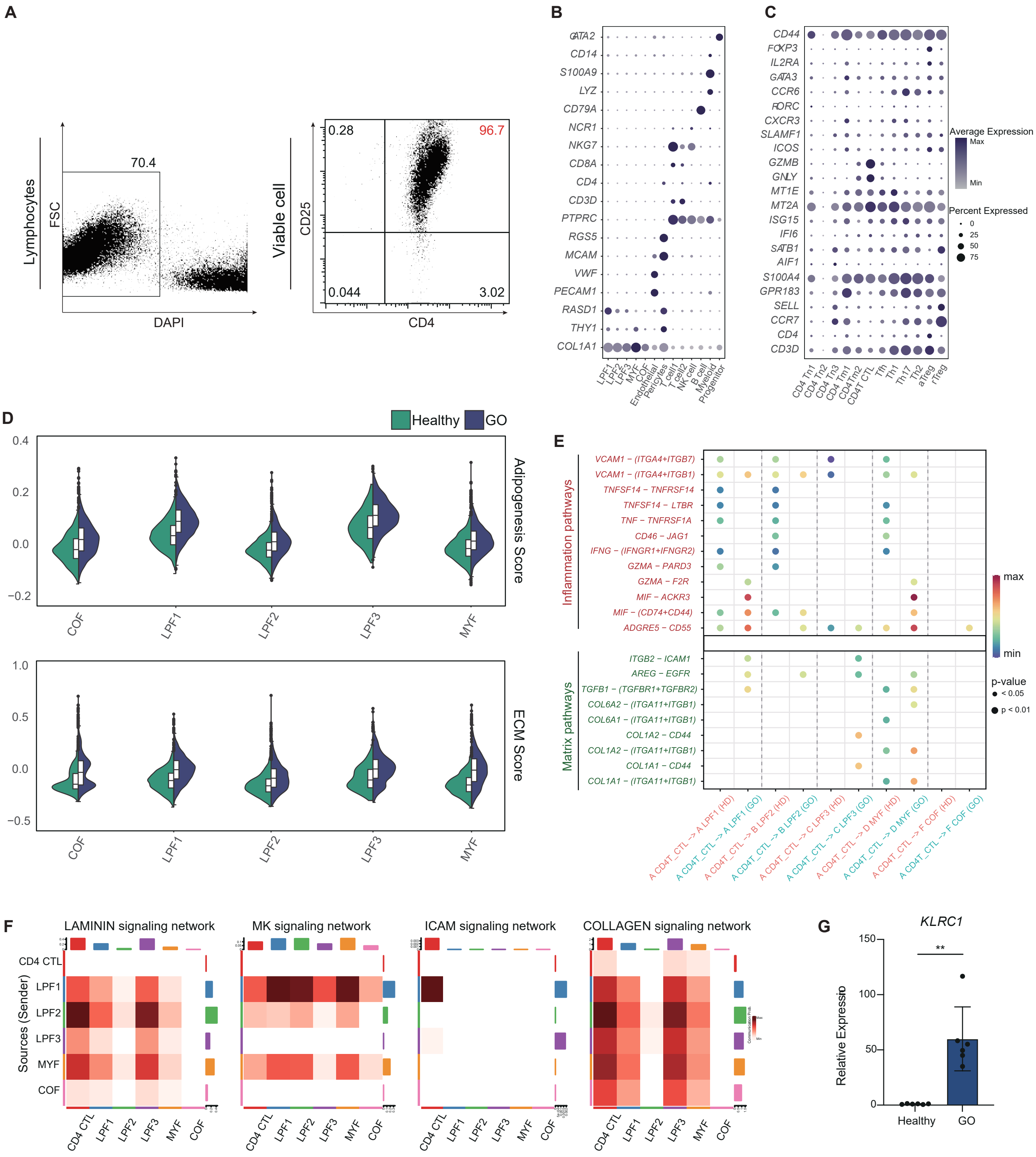

A
Lymphocytes
FSC
70.4
DAPI
Viable cell
CD25
0.28
96.7
0.044
3.02
CD4
B
GATA2
CD14
S100A9
LYZ
CD79A
NCR1
NKG7
CD8A
CD4
CD3D
PTPRC
RGS5
MCAM
VWF
PECAM1
RASD1
THY1
COL1A1
LPF1
LPF2
LPF3
MYF
COF
Endothelial
Pericytes
T cell1
T cell2
NK cell
B cell
Myeloid
Progenitor
C
CD44
FOXP3
IL2RA
GATA3
CCR6
RORC
CXCR3
SLAMF1
ICOS
GZMB
GNLY
MT1E
MT2A
ISG15
IFI6
SATB1
AIF1
S100A4
GPR183
SELL
CCR7
CD4
CD3D
CD4 Tn1
CD4 Tn2
CD4 Tn3
CD4 Tm1
CD4Tm2
CD4T CTL
Tfh
Th1
Th17
Th2
aTreg
rTreg
Average Expression
Max
Min
Percent Expressed
0
25
50
75
D
Healthy
GO
Adipogenesis Score
ECM Score
COF
LPF1
LPF2
LPF3
MYF
E
Inflammation pathways
VCAM1 − (ITGA4+ITGB7)
VCAM1 − (ITGA4+ITGB1)
TNFSF14 − TNFRSF14
TNFSF14 − LTBR
TNF − TNFRSF1A
CD46 − JAG1
IFNG − (IFNGR1+IFNGR2)
GZMA − PARD3
GZMA − F2R
MIF − ACKR3
MIF − (CD74+CD44)
ADGRE5 − CD55
Matrix pathways
ITGB2 − ICAM1
AREG − EGFR
TGFB1 − (TGFBR1+TGFBR2)
COL6A2 − (ITGA11+ITGB1)
COL6A1 − (ITGA11+ITGB1)
COL1A2 − CD44
COL1A2 − (ITGA11+ITGB1)
COL1A1 − CD44
COL1A1 − (ITGA11+ITGB1)
max
min
p-value
< 0.05
p < 0.01
A CD4T_CTL −> A LPF1 (HD)
A CD4T_CTL −> A LPF1 (GO)
A CD4T_CTL −> B LPF2 (HD)
A CD4T_CTL −> B LPF2 (GO)
A CD4T_CTL −> C LPF3 (HD)
A CD4T_CTL −> C LPF3 (GO)
A CD4T_CTL −> D MYF (HD)
A CD4T_CTL −> D MYF (GO)
A CD4T_CTL −> F COF (HD)
A CD4T_CTL −> F COF (GO)
F
LAMININ signaling network
MK signaling network
ICAM signaling network
COLLAGEN signaling network
Sources (Sender)
CD4 CTL
LPF1
LPF2
LPF3
MYF
COF
G
KLRC1
Relative Expressio
150
100
50
0
**
Healthy
GO

Supplemental Figure 11. Analysis related to orbital matrix remodeling in GO mouse model
(A) Flow cytometry plots displayed cell viability and the success rate of polarization of CD4+ T cells into Tregs in vitro.
(B) Bubble plot depicted characteristic markers of major subtypes in the orbital tissues of Healthy donors and GO patients.
(C) Bubble plot illustrated characteristic markers of integrated subtypes of CD4 T cells between orbital tissues and PBMC from Healthy donors and GO patients.
(D) Expression scores of Adipogenesis and ECM functional gene sets in all fibroblast subtypes of Healthy donors and GO patients.
(E) Differential signaling pathways between the Healthy and GO groups.
(F) Interaction strength of LAMININ, MK, ICAM, and COLLAGEN signaling network interactions between CD4 CTL and various fibroblast subtypes.
(G) The qRT-PCR bar chart displayed the expression of specific genes in Tregs after treatment with TSHR and TNF-α. Data are represented as the median [IQR]. **$P < 0.001$ by Mann-Whitney U test. The experiments were repeated 3 times.
